# Supplementary material for: Coulomb pre-stress and fault bends are ignored yet vital factors for earthquake triggering and hazard
Source: Nat Commun. 2019 Jun 21;10:2744. doi: 10.1038/s41467-019-10520-6 (PMC6588554; doi:10.1038/s41467-019-10520-6)
Supplement: Supplementary file 2 — Description of Additional Supplementary Files [file 41467_2019_10520_MOESM2_ESM.pdf]

Description of Additional Supplementary Files

File Name: Supplementary Data 1
Description: Coseismic CST from each Mw>5.5 historical earthquake that has occurred since 1349 A.D.. The date, fault name that ruptures next and the magnitude of the earthquake are given on each figure. The surface traces of active faults are shown in green, the trace of the fault that ruptures is shown in yellow, the trace of the next fault that ruptures is shown in pink. White regions show the extent of the brittle faults at depth where negligible stress has been transferred.

File Name: Supplementary Data 2
Description : Coulomb pre-stress, comprised of interseismic and coseismic (from historical earthquakes) CST calculated prior to each Mw>5.5 earthquake in the historical record. The date, fault name that ruptures next and the magnitude of the earthquake are given on each figure. The surface traces of active faults are shown in green, the trace of the next fault that ruptures is shown in pink. White regions show the extent of the brittle faults at depth where negligible stress has been transferred. Note the non-linear colour scale.

File Name: Supplementary Data 3
Description : Testing the sensitivity of the coseismic CST transferred to receiver faults in relation to the slip distribution. The Coulomb stress transferred and the corresponding slip distribution on the Norcia fault are shown for five models. CSM- Costa-San Martino, LAG- Laga, LEO- Leonessa, MAR-Martana, MLS- Mt. Le Scalette, MOT- Montereale, MTV- Mt. Vettore, NOR- Norcia, TER- Terni, UMV-Umbra Valley. a. Location of maximum slip in the centre of the fault plane. b. Location of maximum slip skewed to 5 km depth and 5km from the north west end of the fault. c. Location of maximum slip skewed to 5 km depth and 5km from the southeast end of the fault. d. Location of maximum slip skewed to 10 km depth and 5km from the north-west end of the fault. e. Location of maximum slip skewed to 10 km depth and 5km from the south-east end of the fault. The pattern of positive and negative stress is relatively consistent across all models, the magnitude of the Coulomb stress transferred differs between the models.

File Name: Supplementary Data 4
Description : An Excel spreadsheet giving the data and analysis of the coseismic and cumulative CST presented in the text and in Figure 4. The spreadsheet is arranged into 5 sheets, the README sheet gives an overview of the other sheets in the file.

File Name: Supplementary Data 4
Description : An Excel spreadsheet giving the data and analysis of the coseismic and cumulative CST presented in the text and in Figure 4. The spreadsheet is arranged into 5 sheets, the README sheet gives an overview of the other sheets in the file.

File Name: Supplementary Data 5
Description : Fieldwork data used herein that have not previously been published.

File Name: Supplementary Video 1
Description : Animation showing the coseismic CST associated with 34 historical earthquakes from 1349 – 2016 A.D. in the central Apennines. UTM coordinates are given (33T zone). The date, causative fault and magnitude of earthquake are given. The surface traces of active faults are shown in green, the trace of the fault that ruptures is shown in yellow, the trace of the next fault that

ruptures is shown in pink. The animation is comprised of the figures that make up Supplementary Data 1.

File Name: Supplementary Video 2
Description : Animation showing the Coulomb pre-stress (comprised of interseismic and coseismic CST) prior to each of the 34 historical earthquakes from 1349 – 2016 A.D. in the central Apennines. UTM coordinates are given (33T zone). The date, causative fault and magnitude of earthquake are given. The surface traces of active faults are shown in green, the trace of the fault that ruptures is shown in yellow, the trace of the next fault that ruptures is shown in pink. The animation is comprised of the figures that make up Supplementary Data 2.
