# Supplementary figures and images for: Coulomb pre-stress and fault bends are ignored yet vital factors for earthquake triggering and hazard
Source: Nat Commun. 2019 Jun 21;10:2744. doi: 10.1038/s41467-019-10520-6 (PMC6588554; doi:10.1038/s41467-019-10520-6)

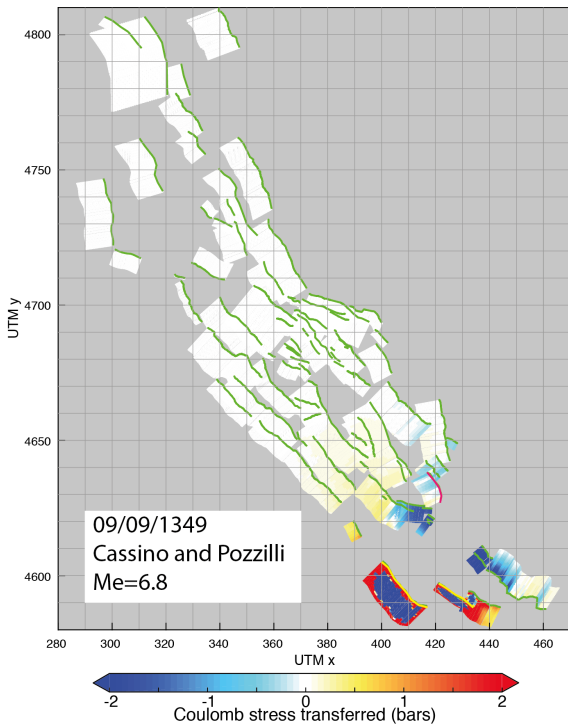

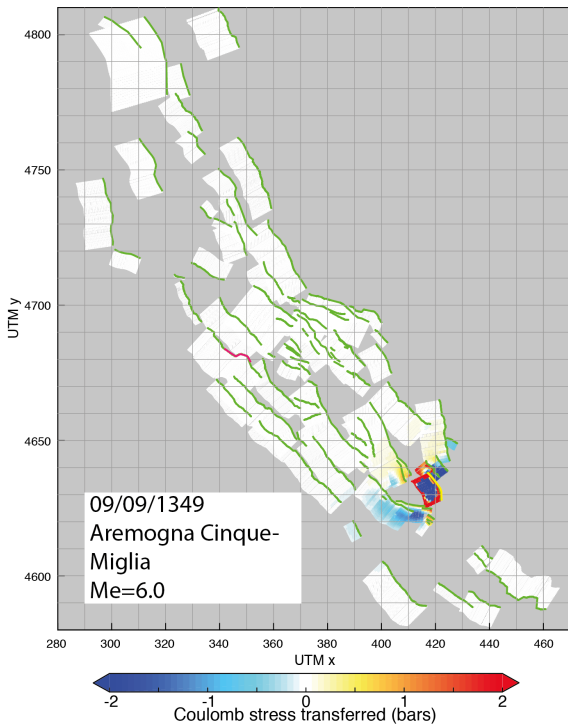

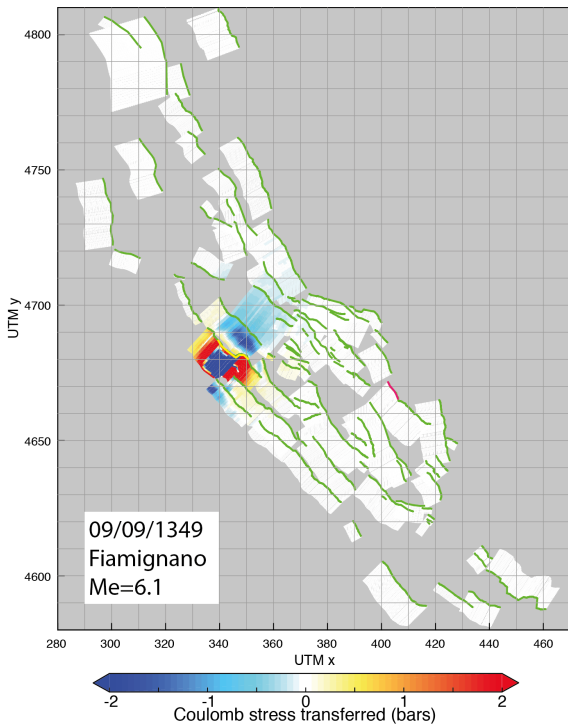

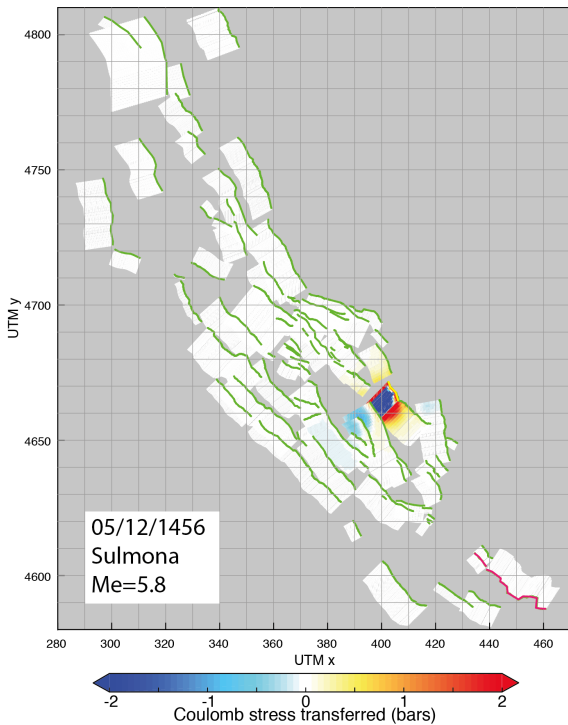

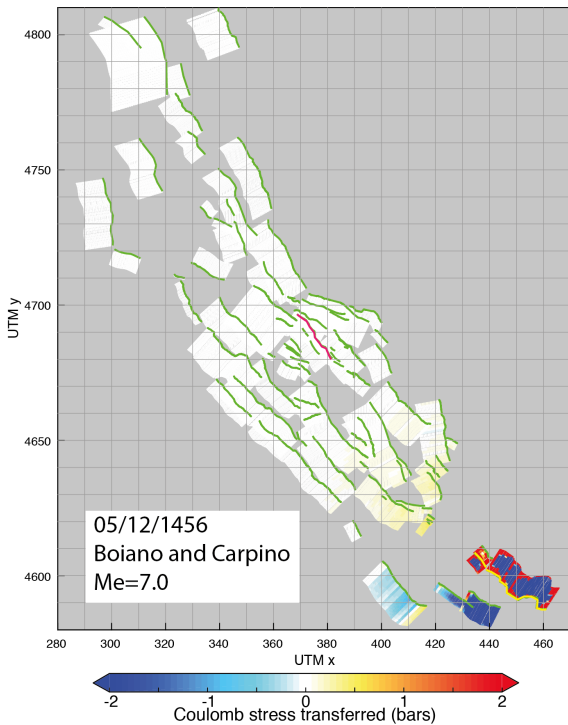

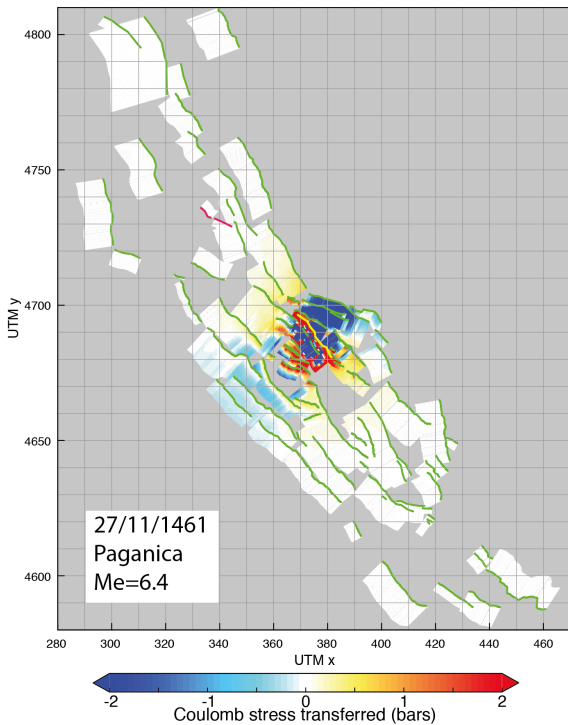

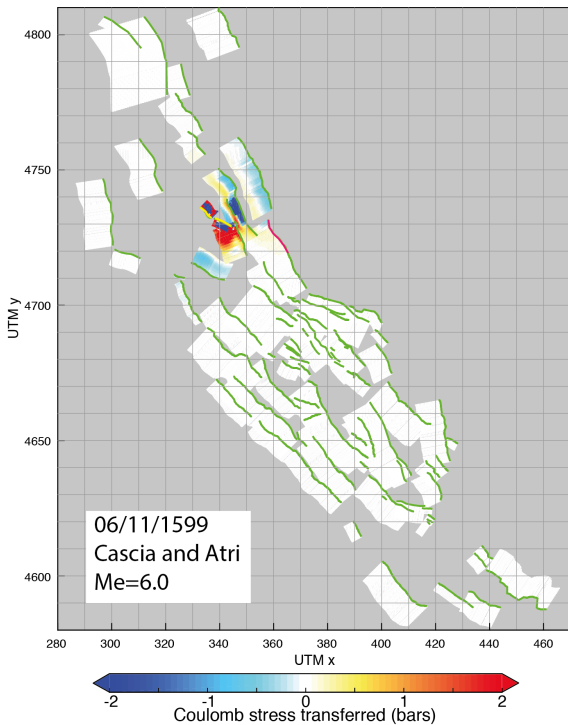

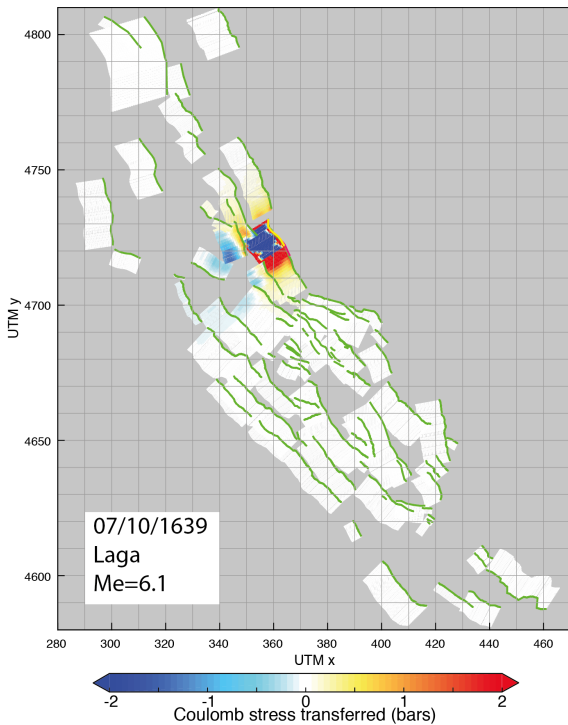

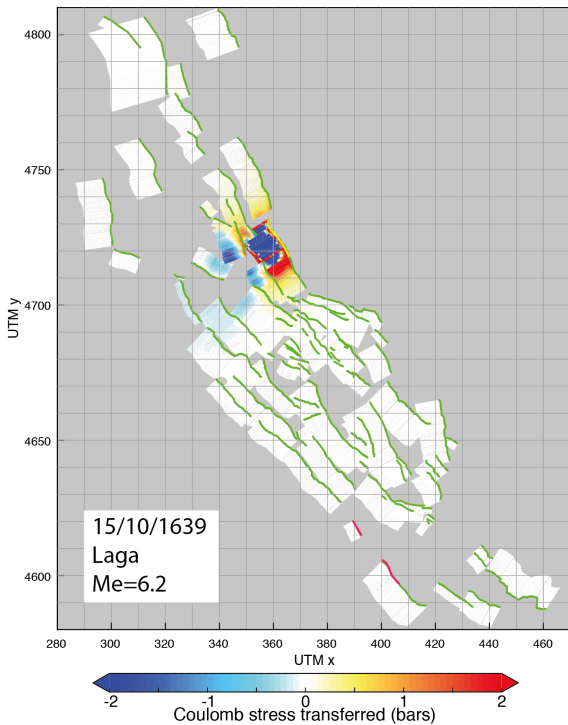

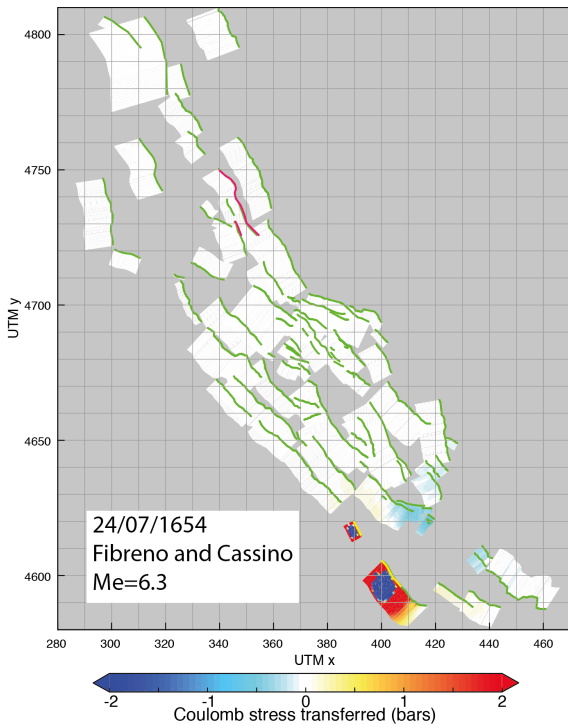

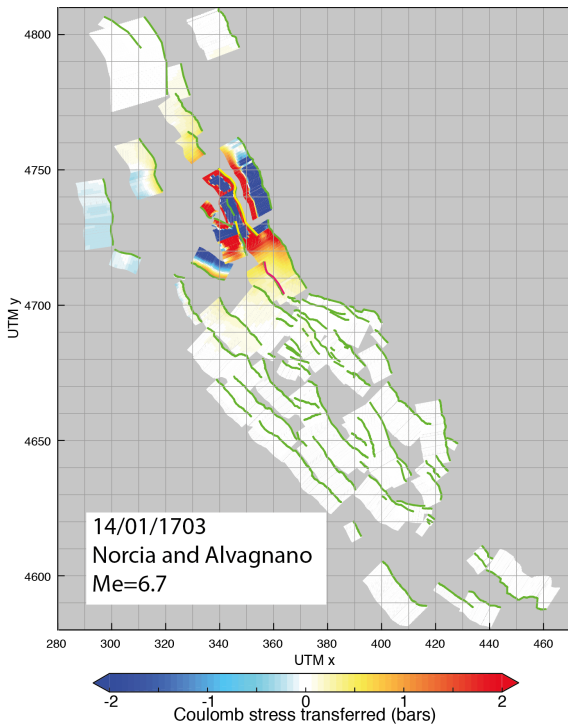

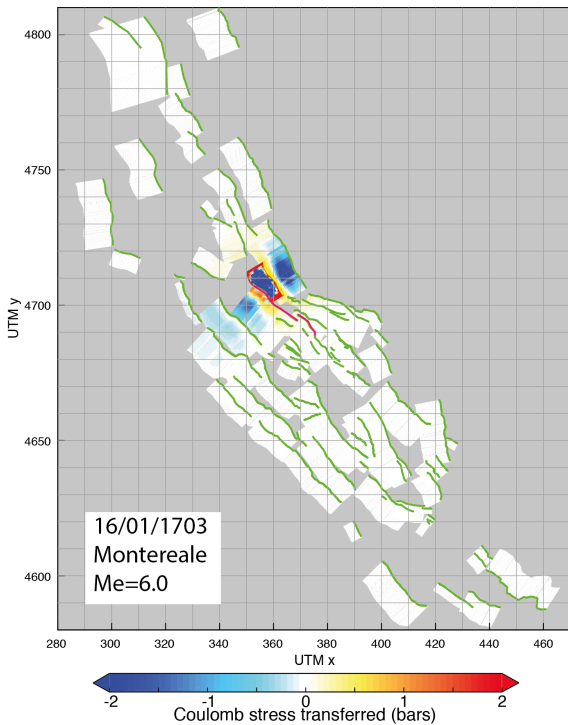

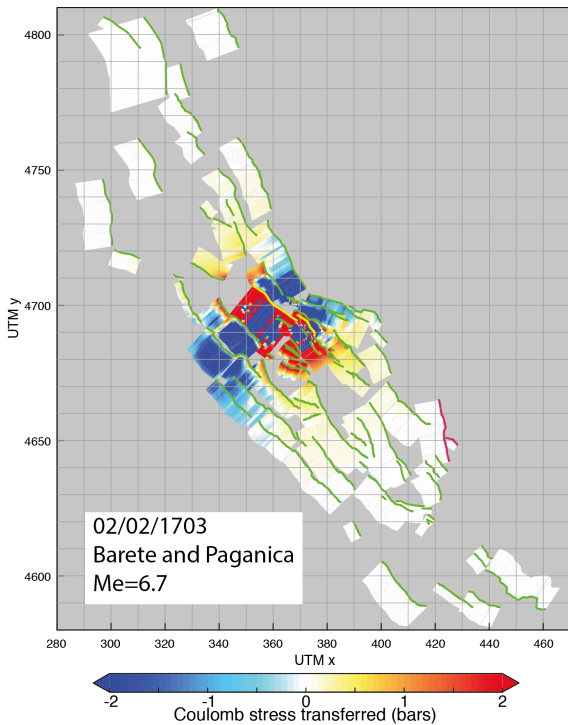

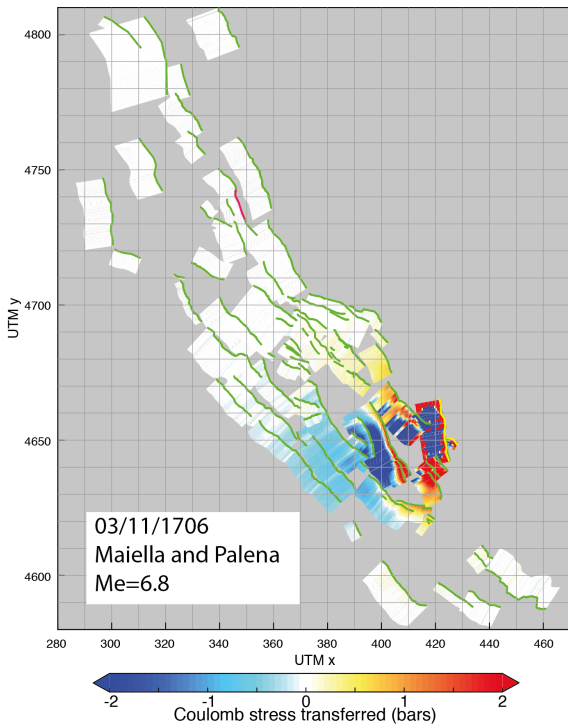

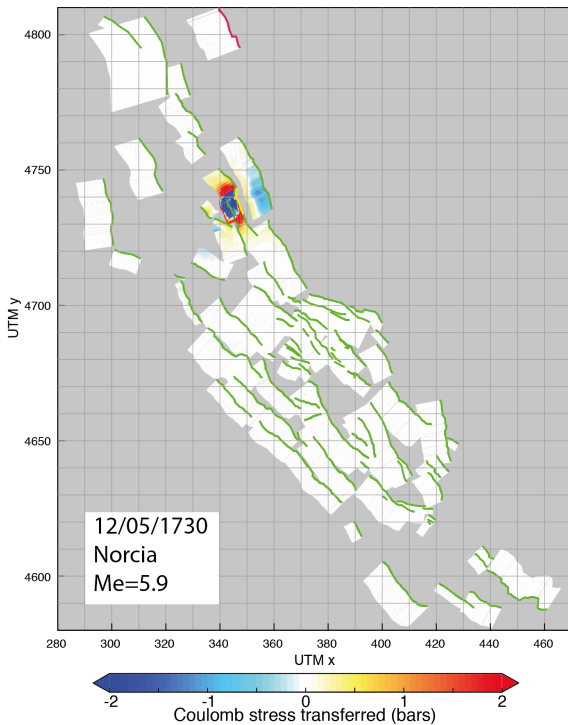

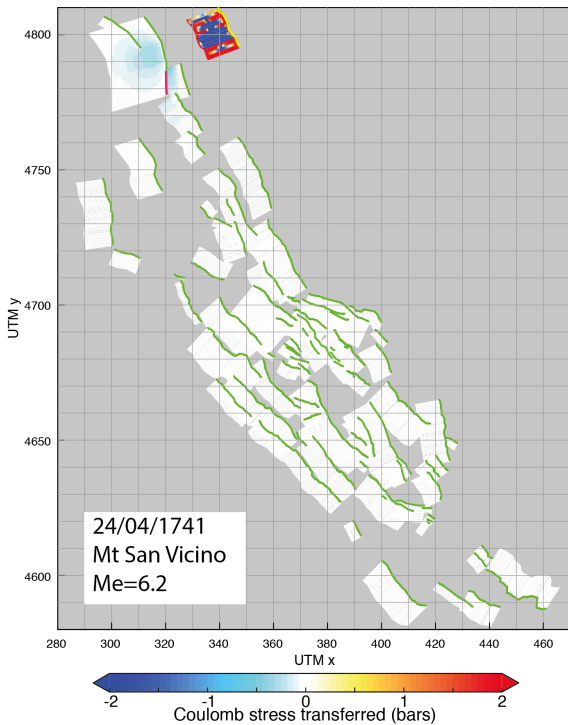

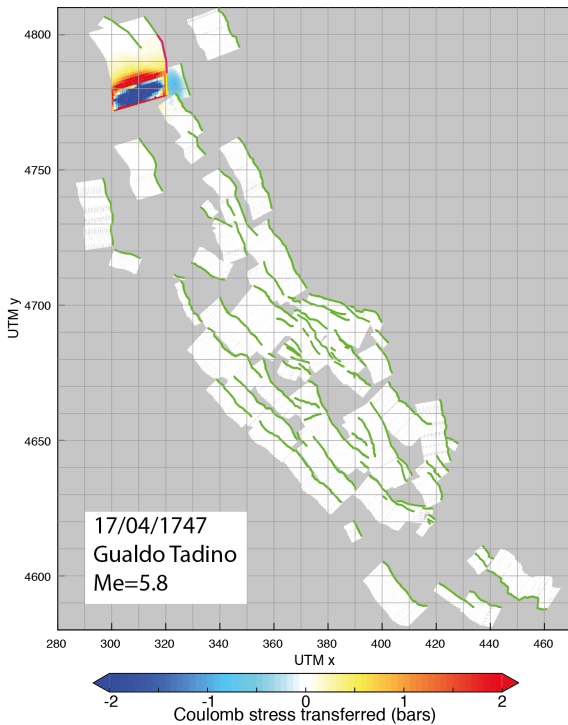

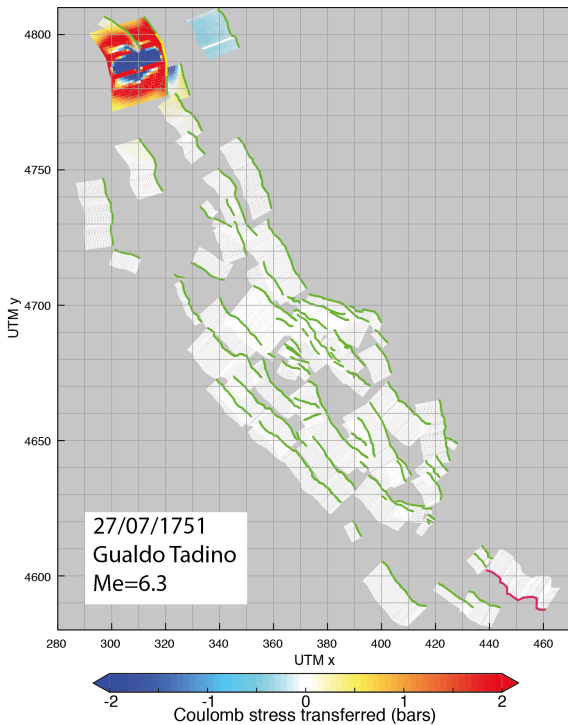

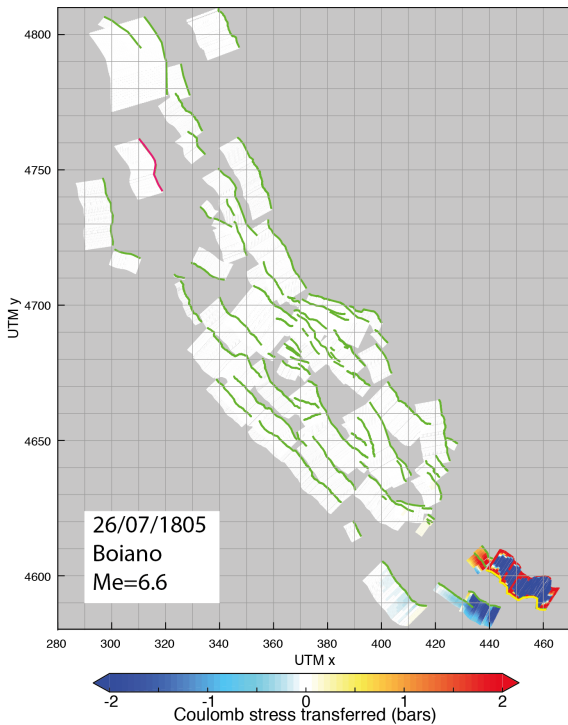

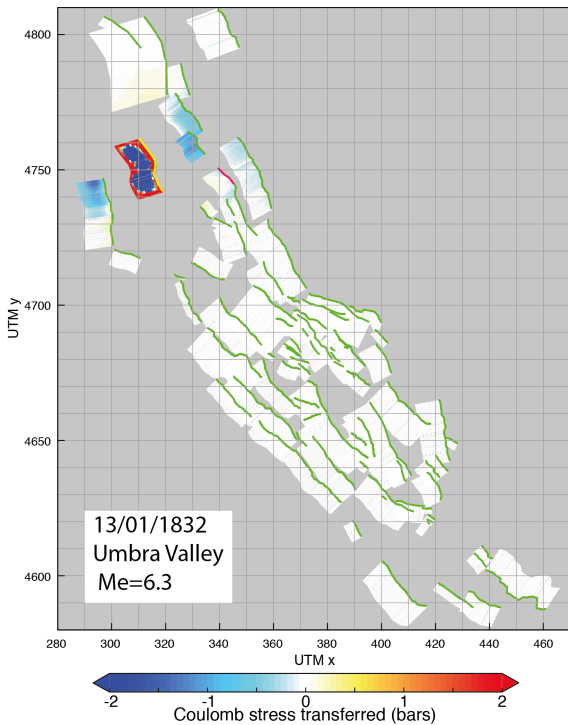

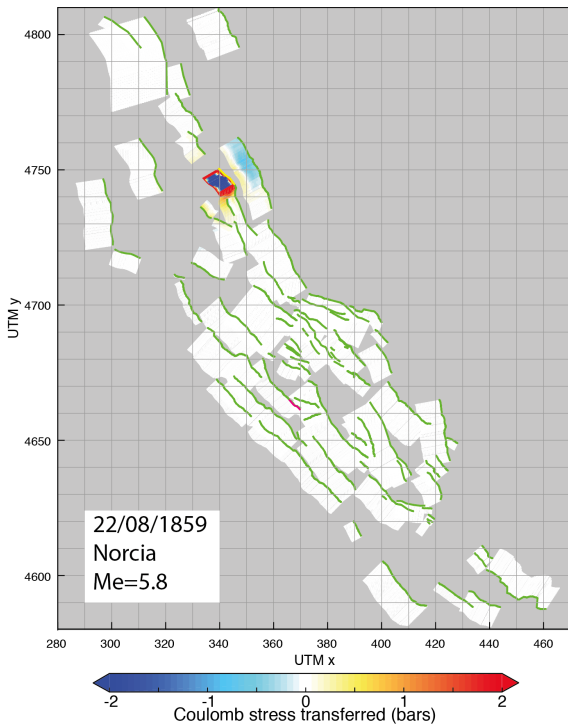

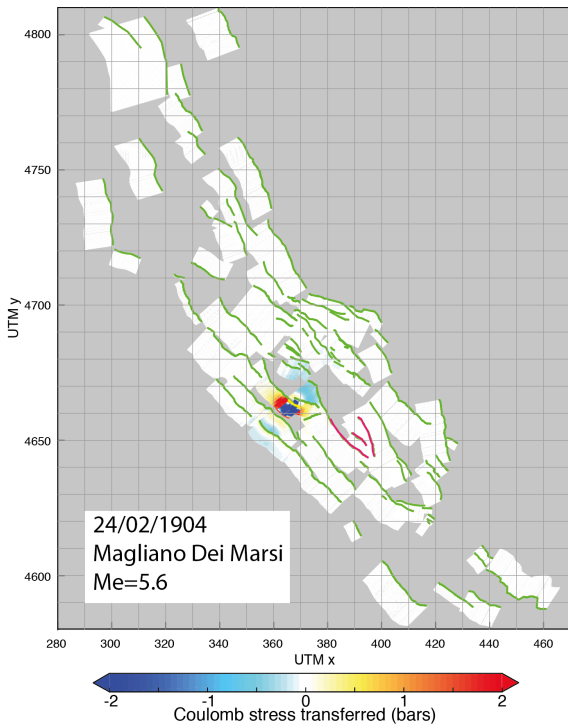

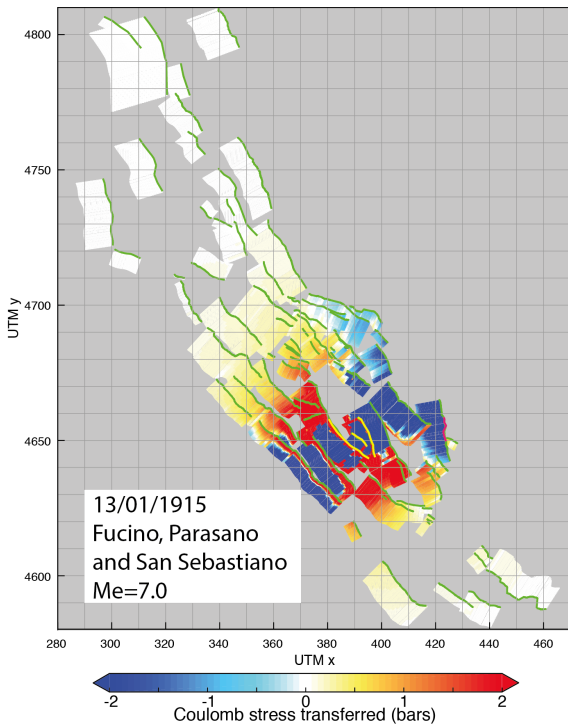

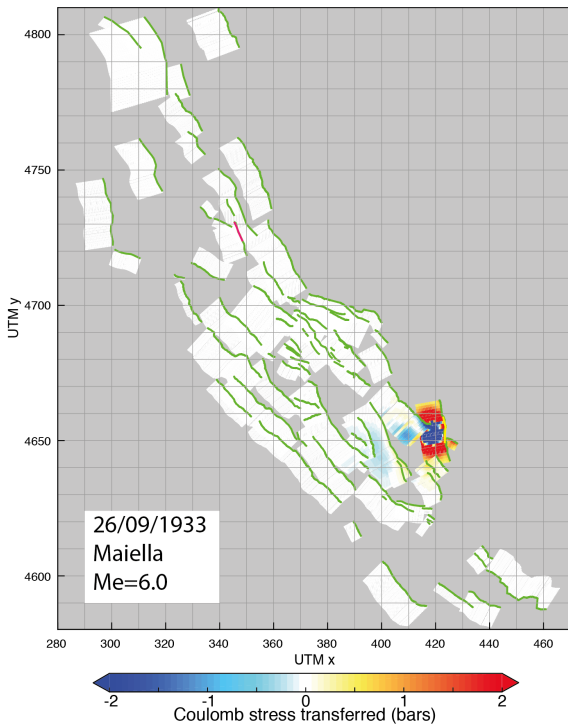

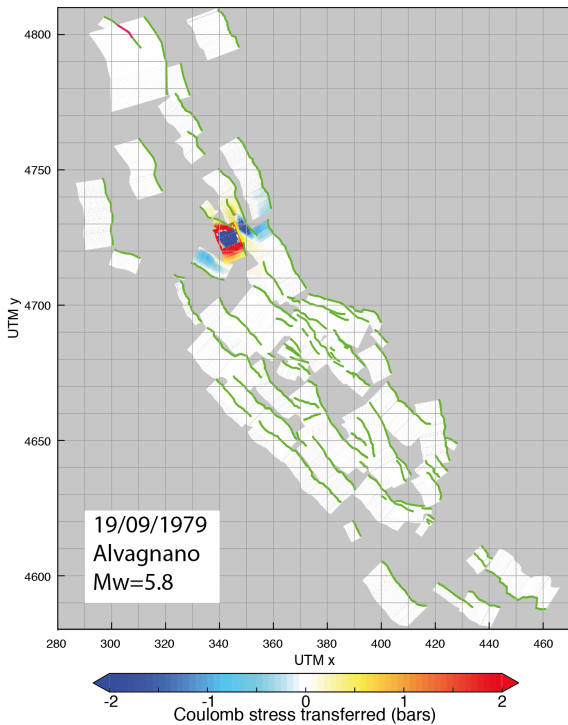

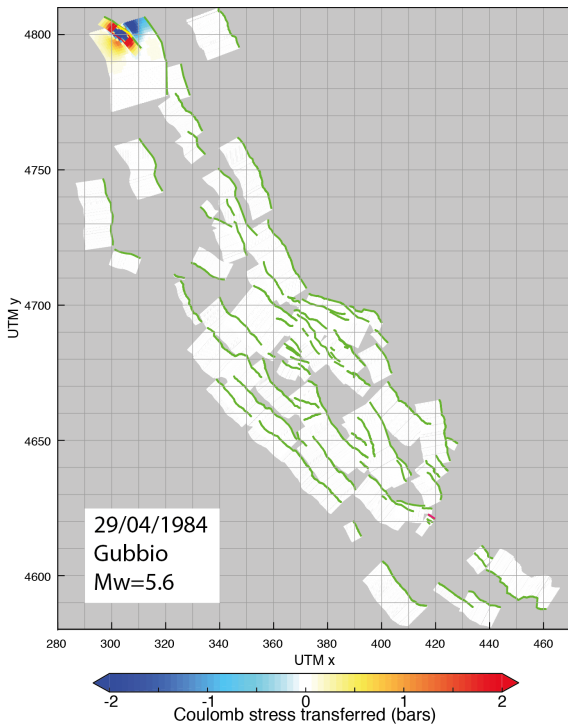

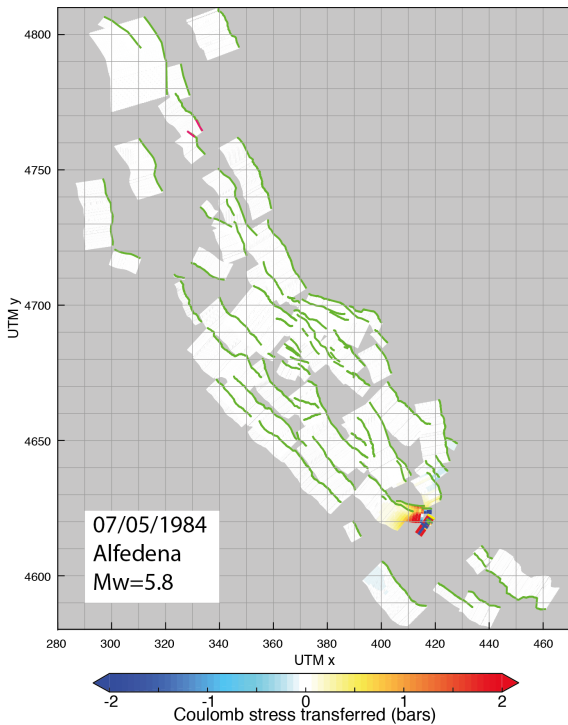

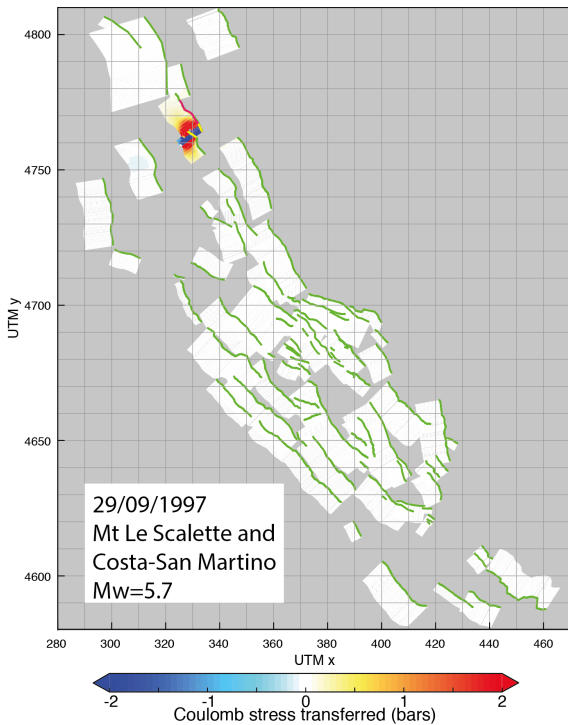

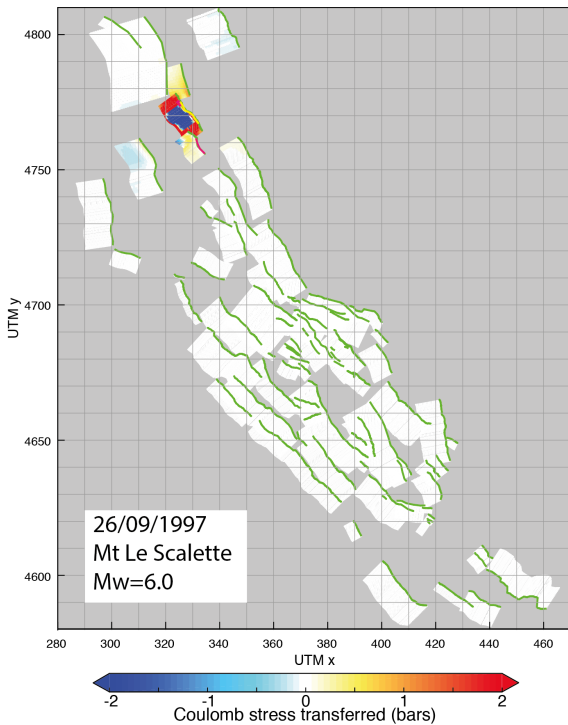

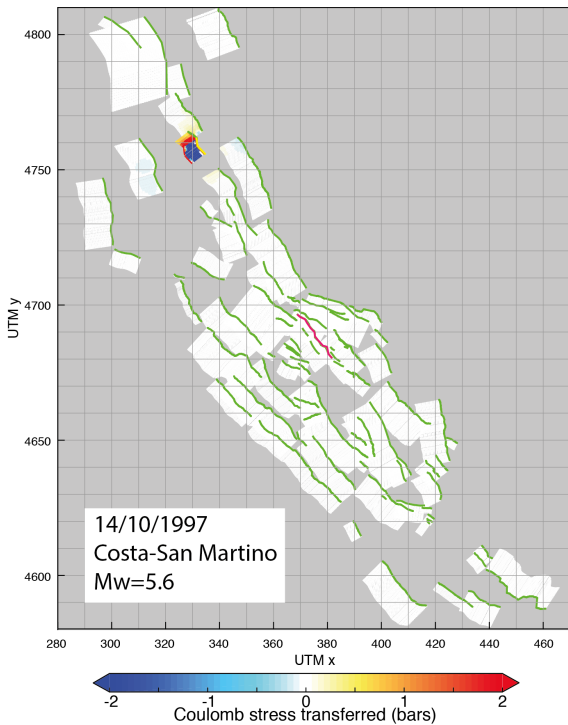

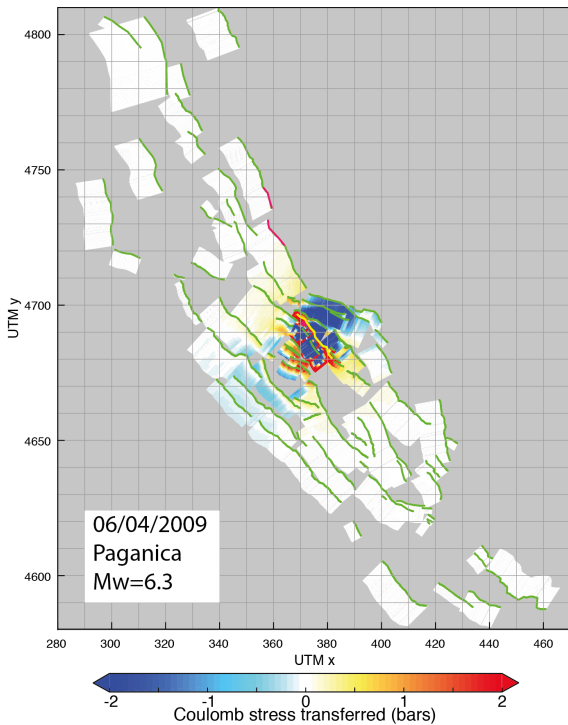

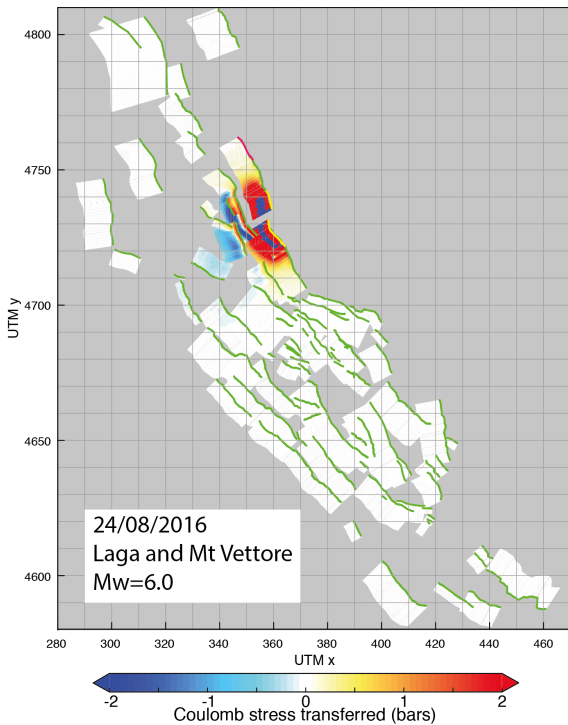

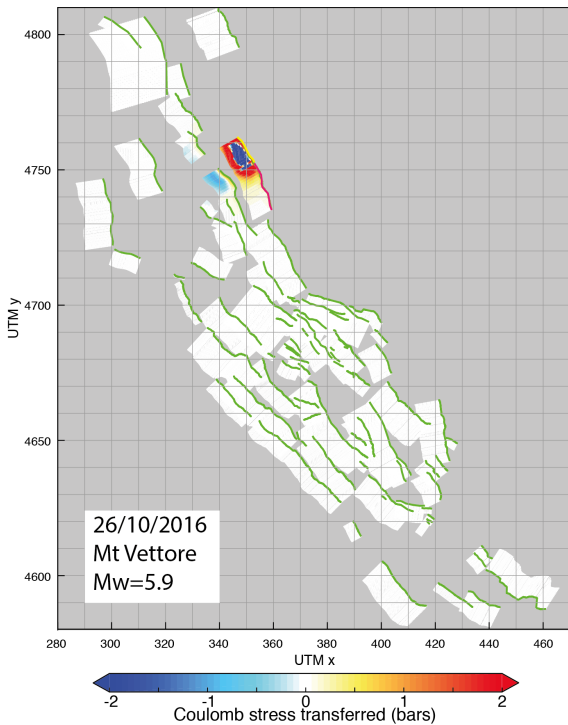

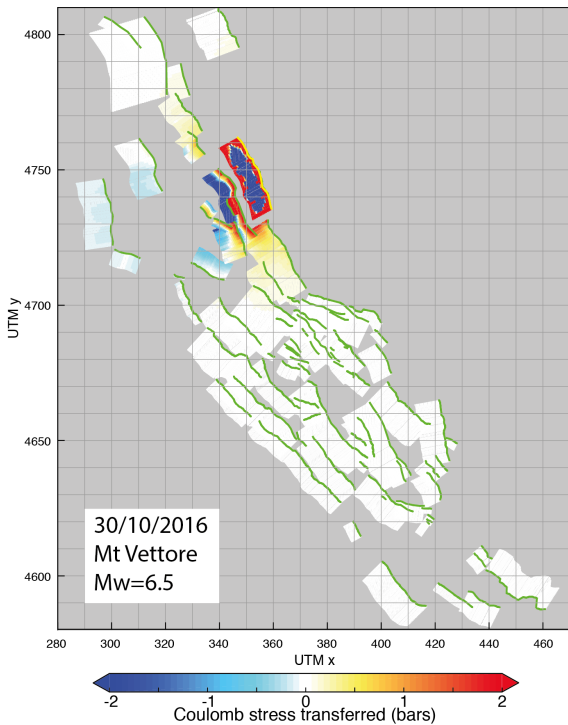

Supplement: Supplementary file 3 — Supplementary Data 1 [file 41467_2019_10520_MOESM3_ESM.pdf]

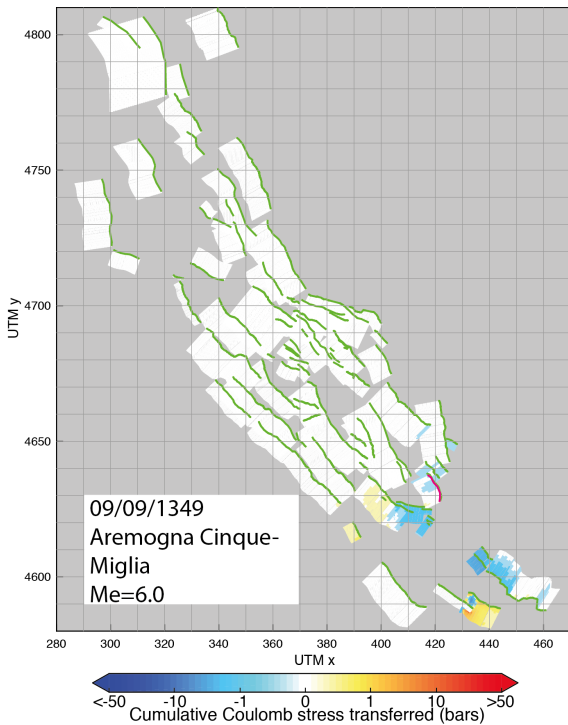

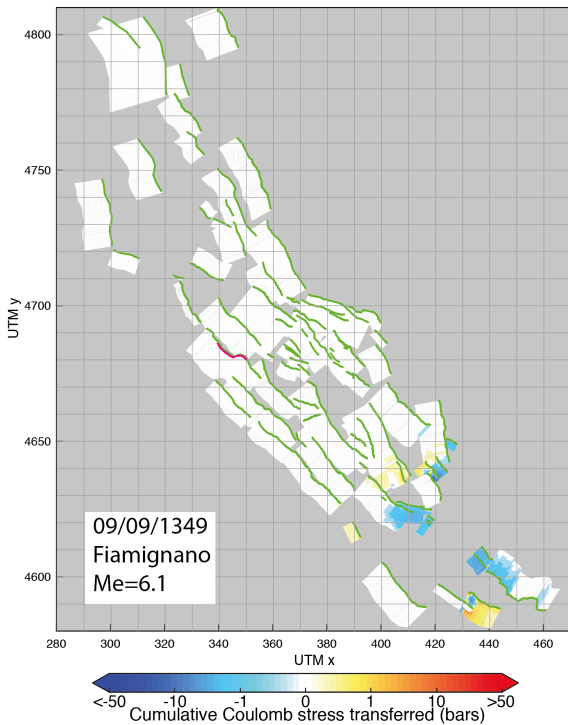

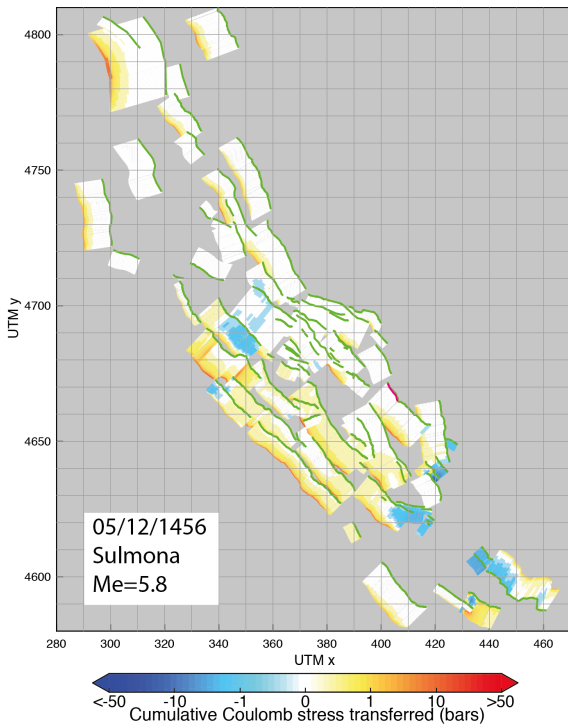

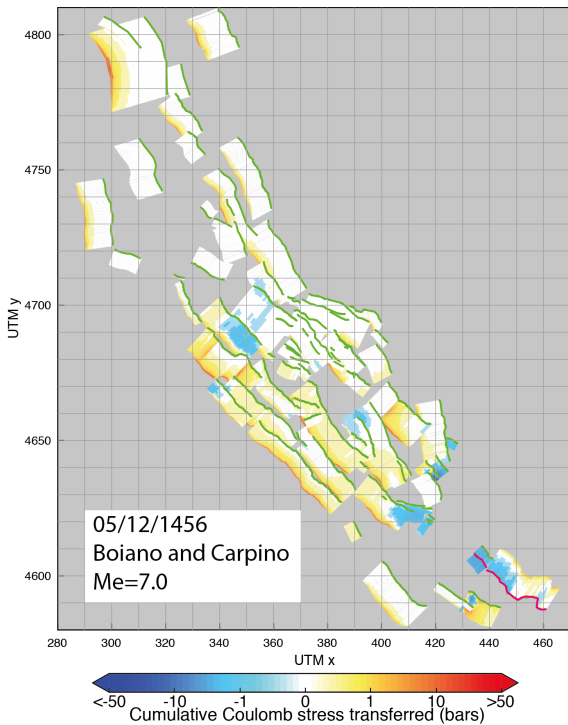

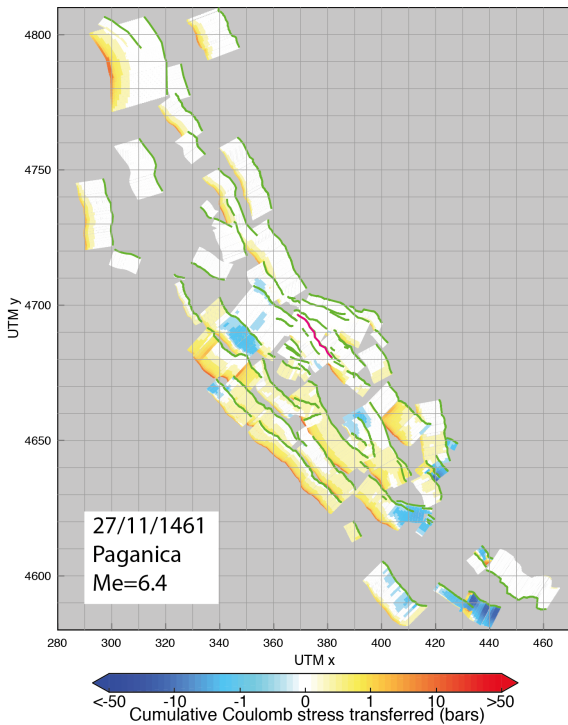

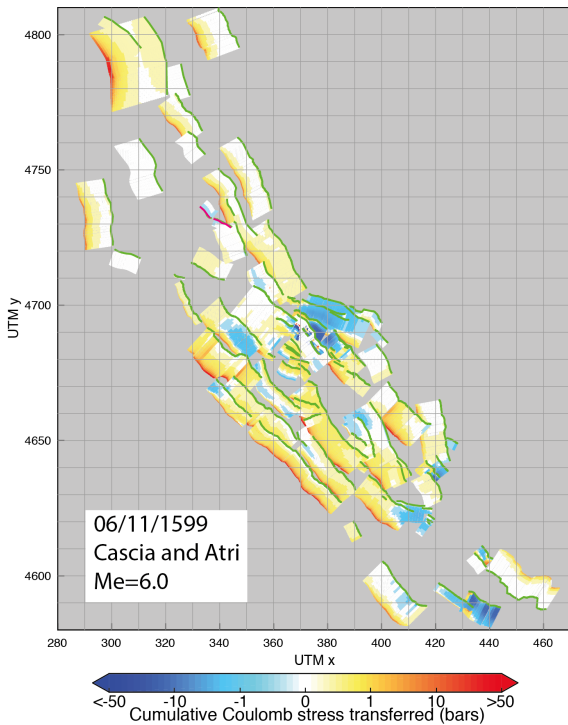

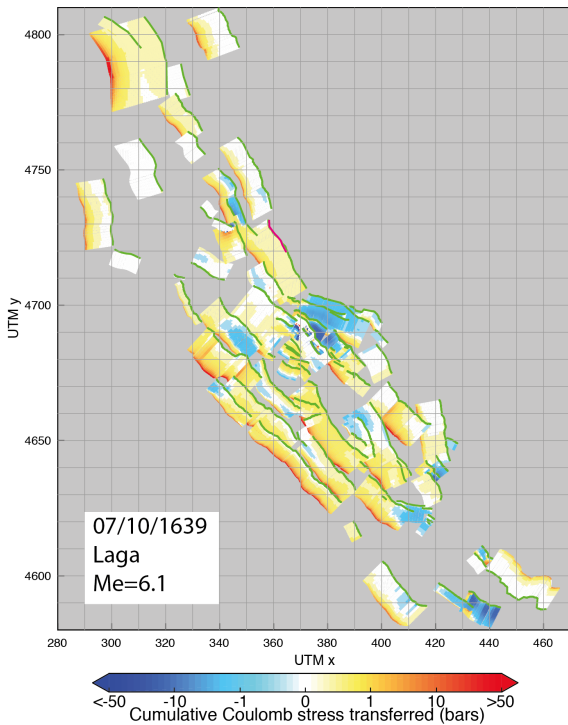

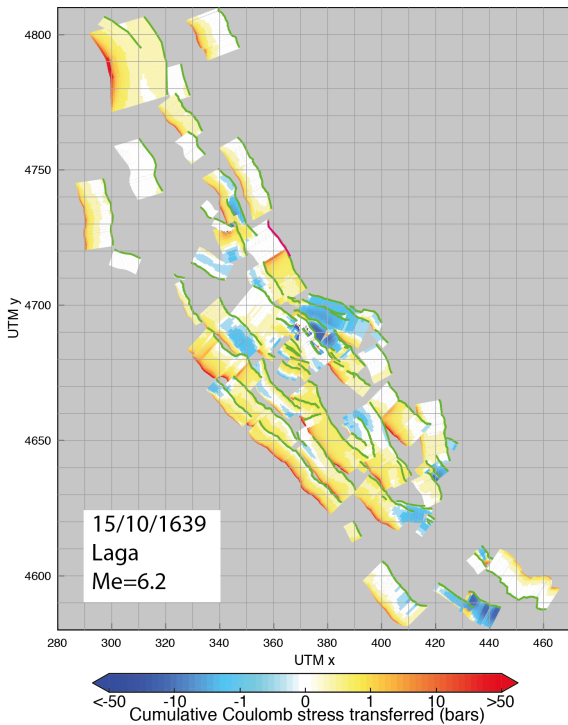

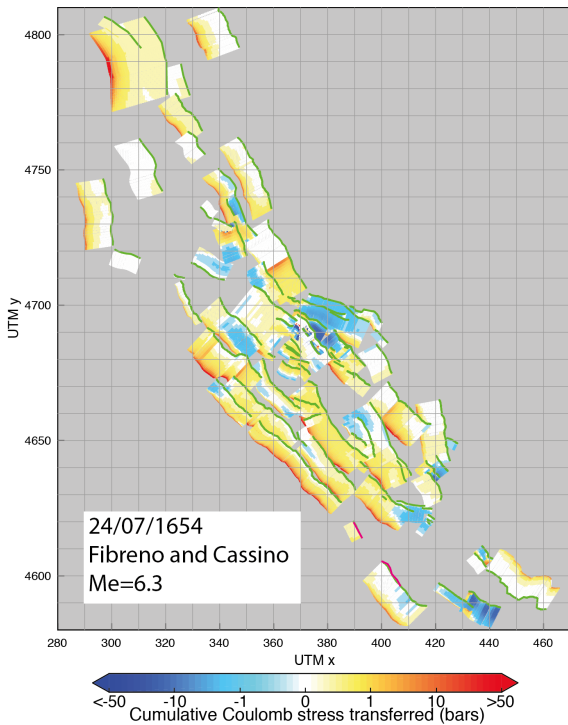

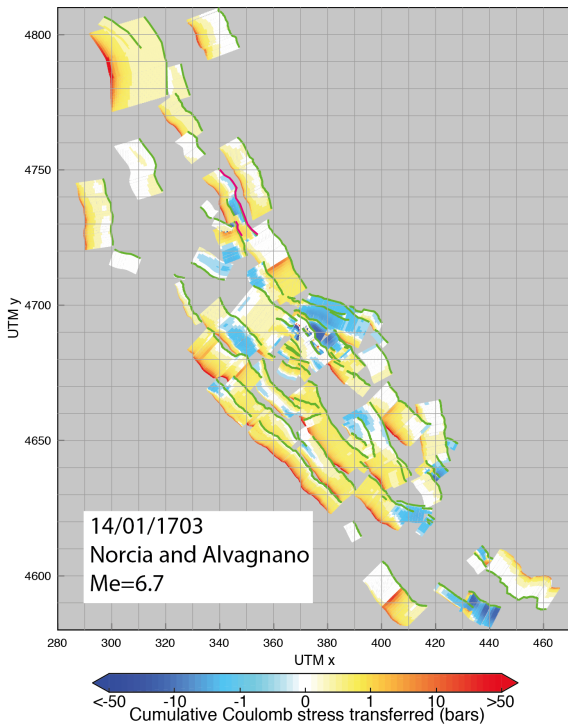

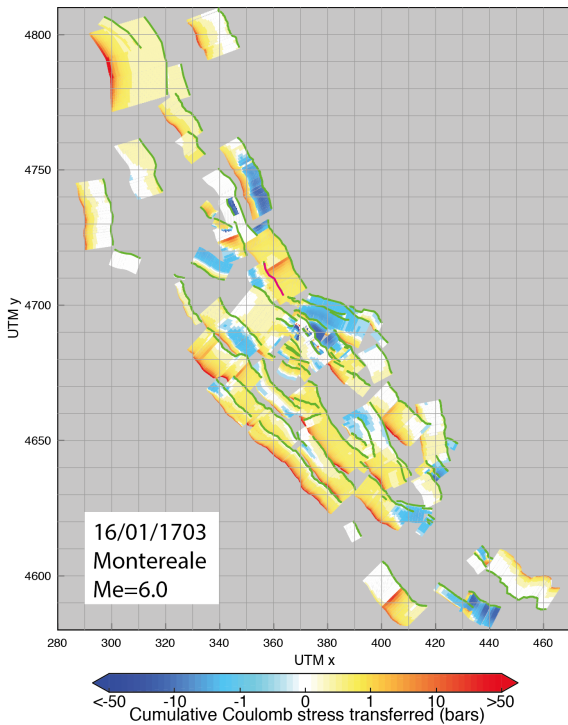

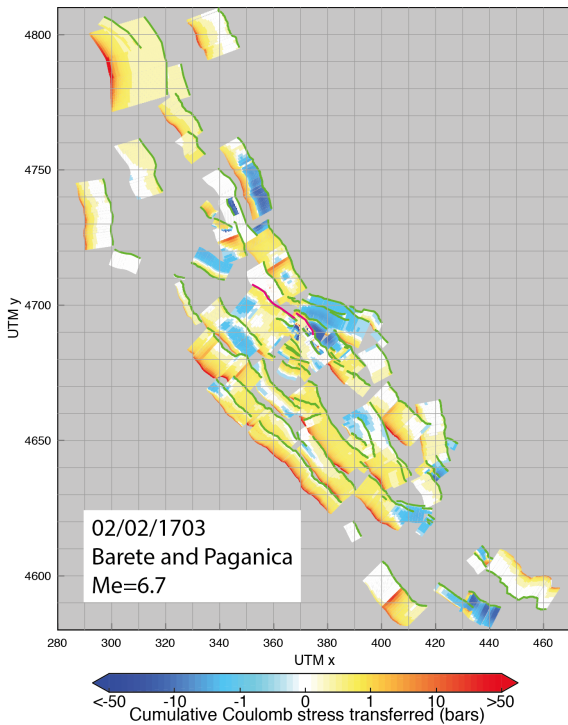

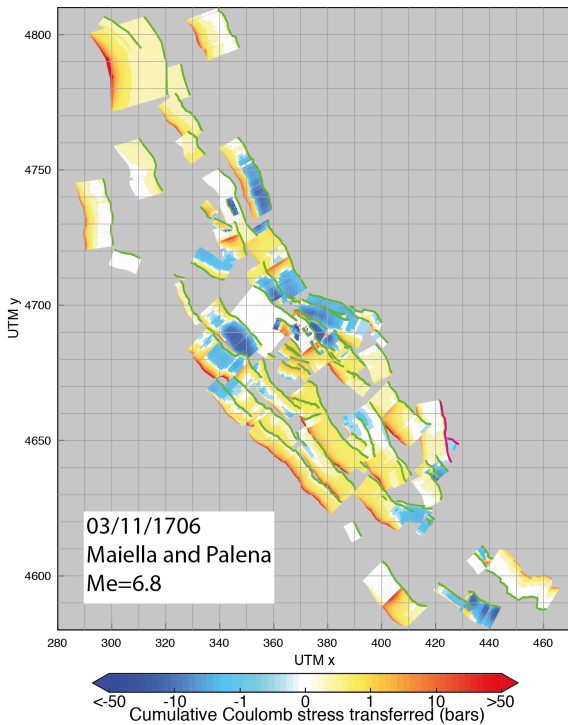

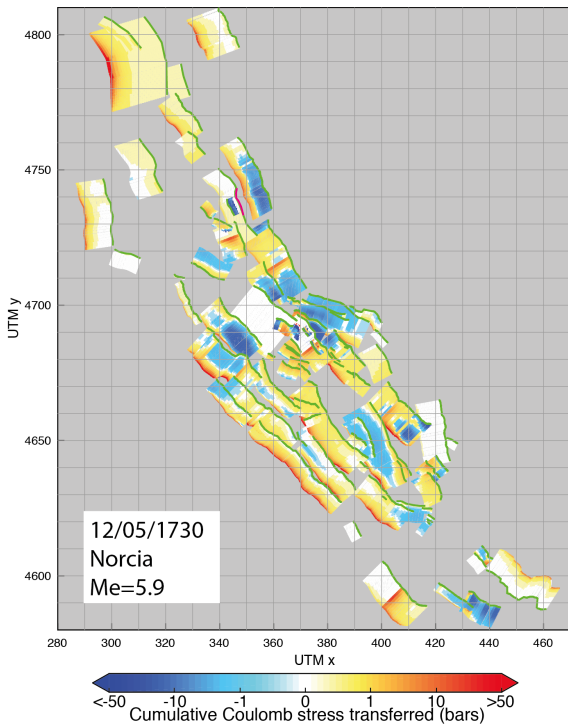

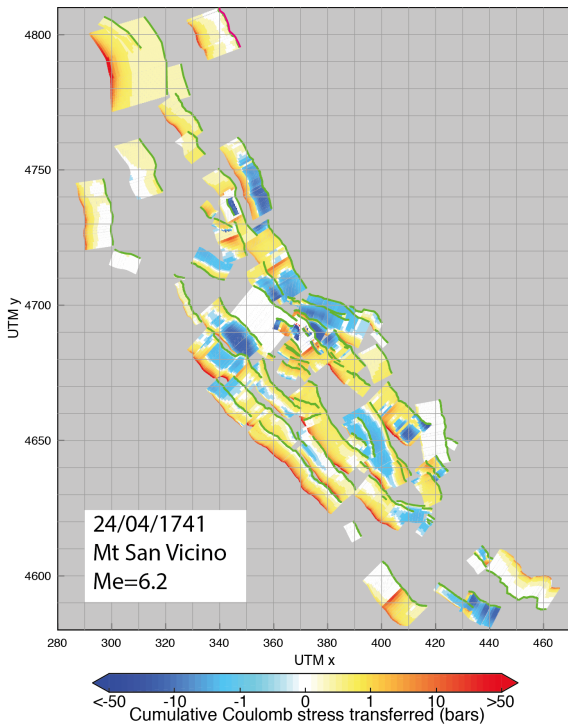

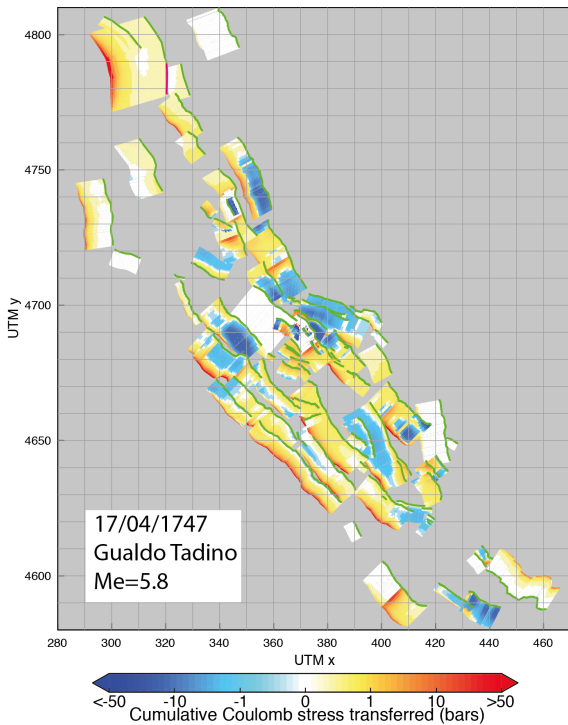

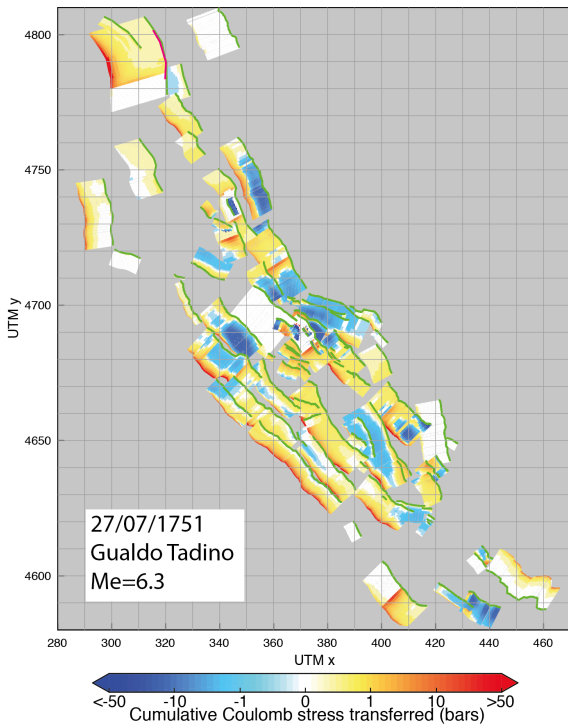

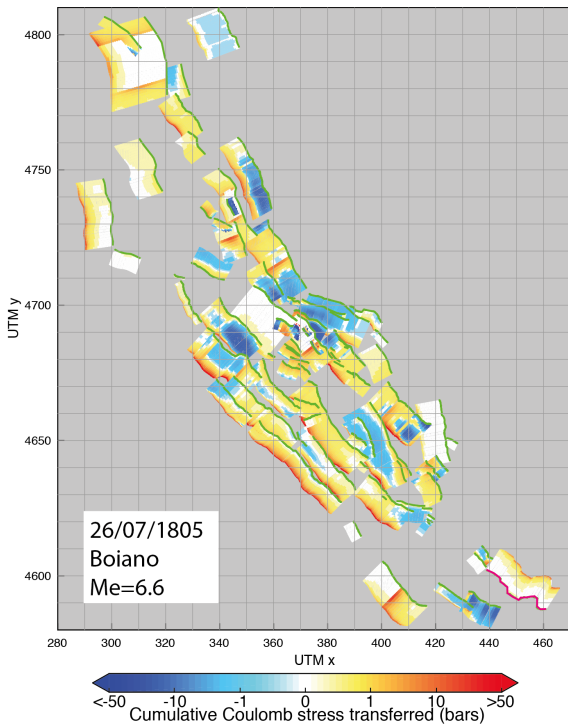

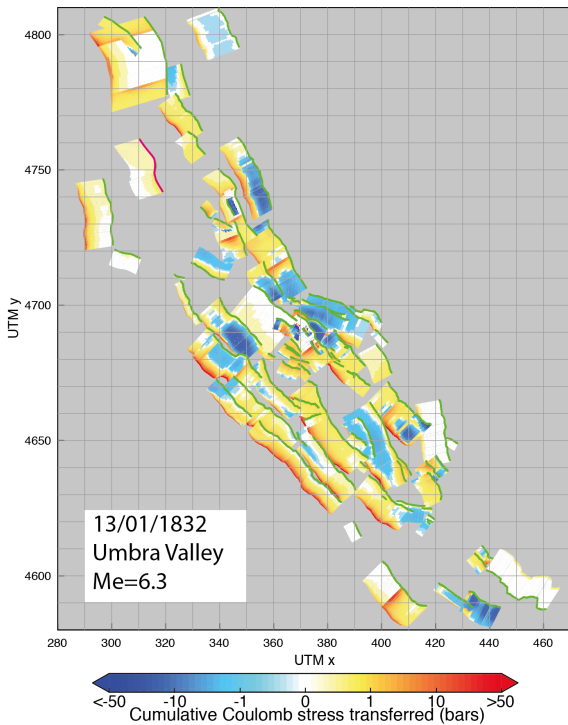

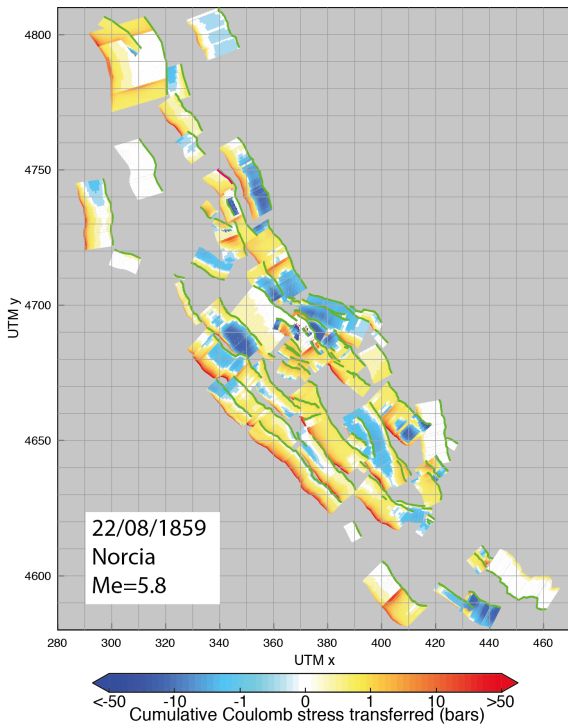

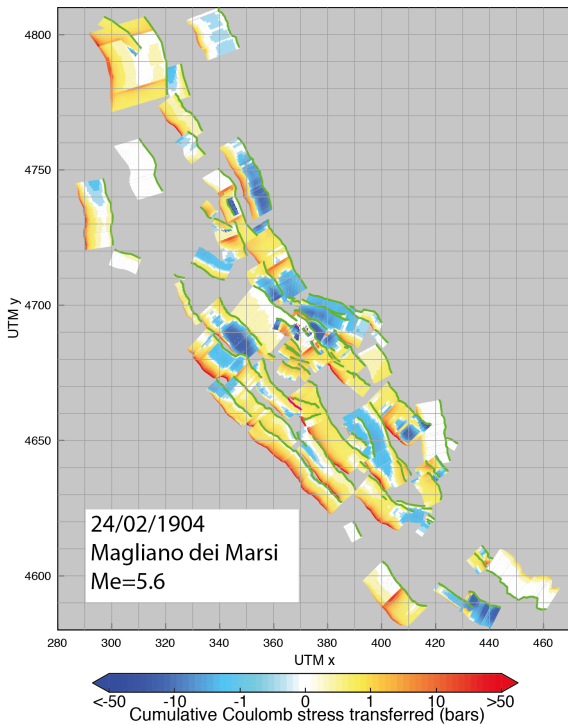

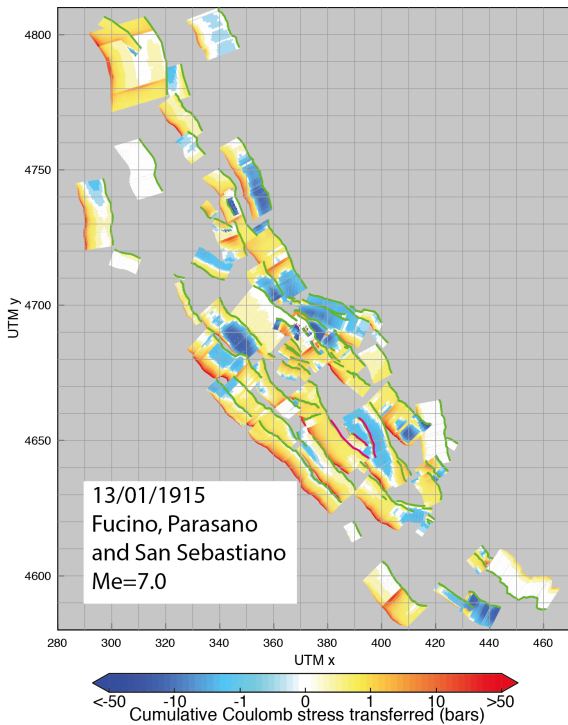

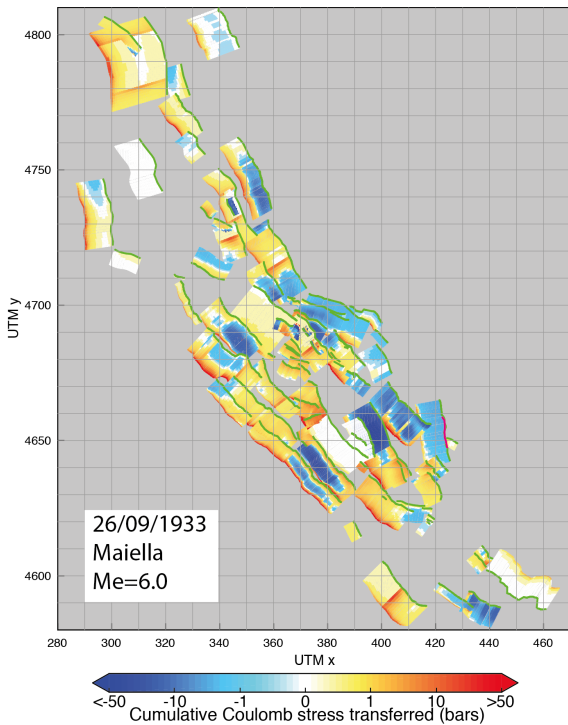

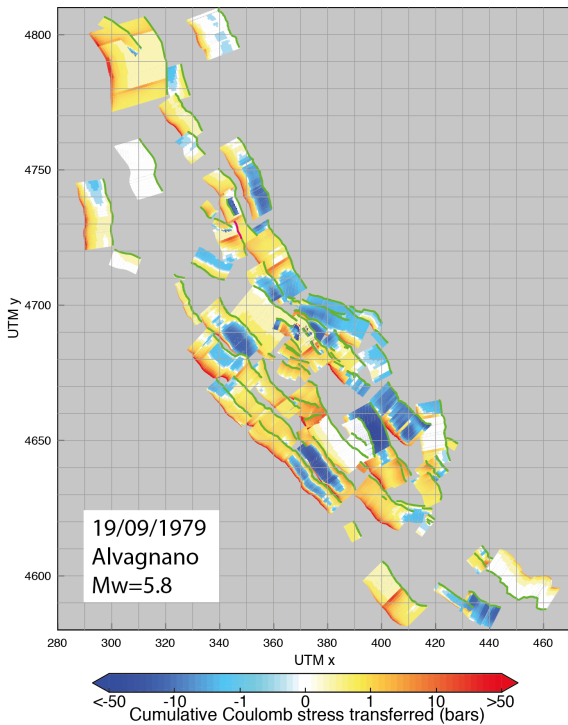

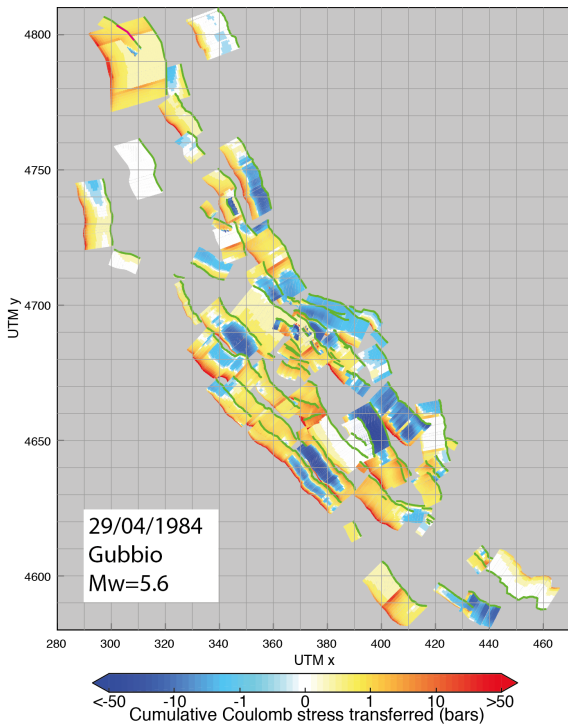

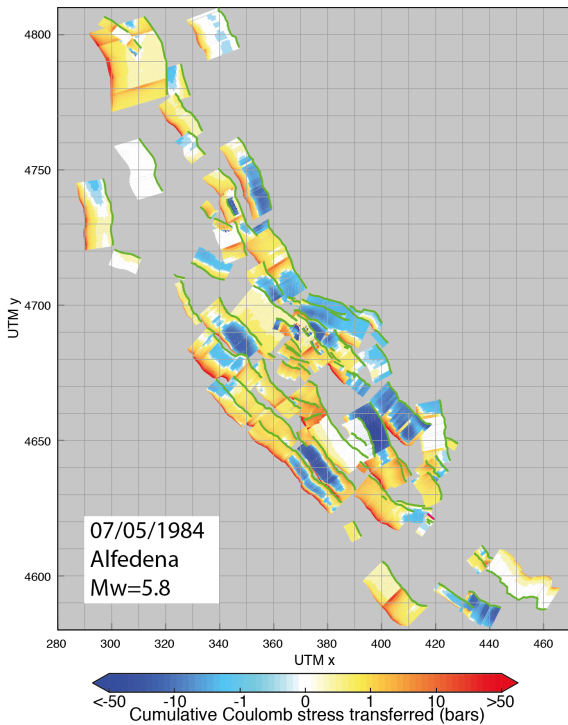

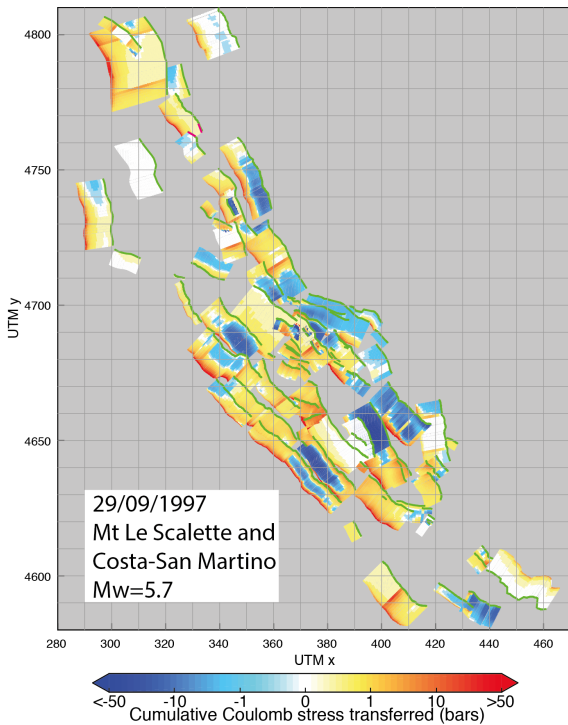

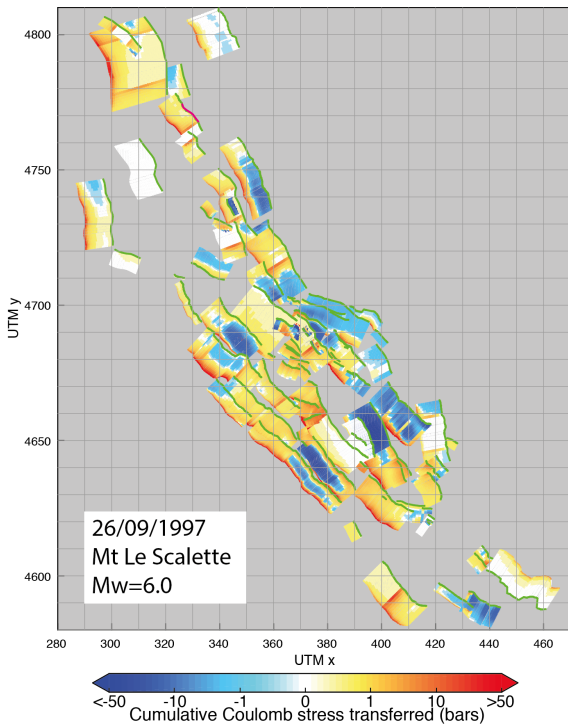

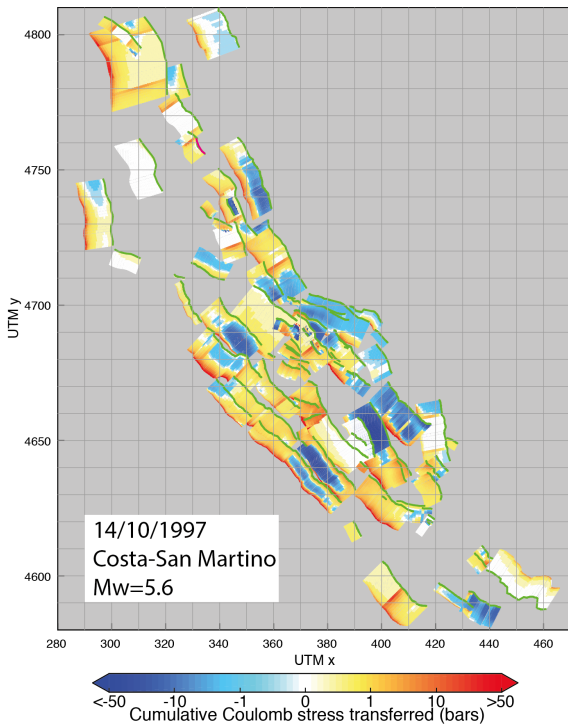

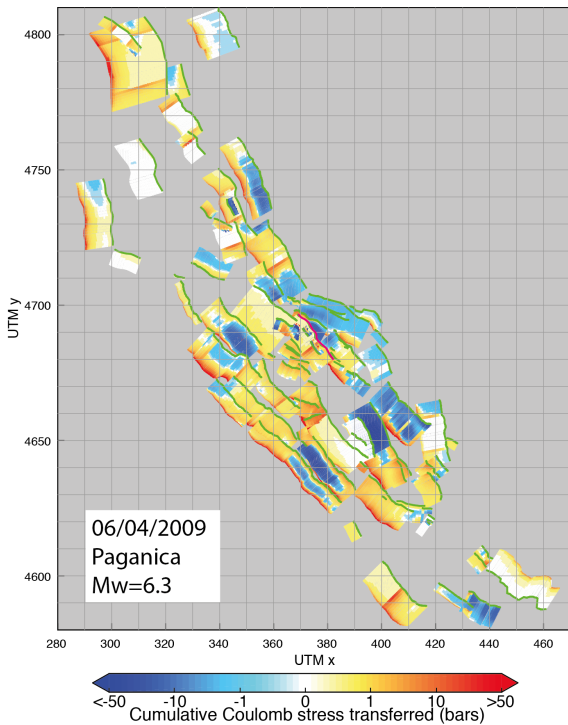

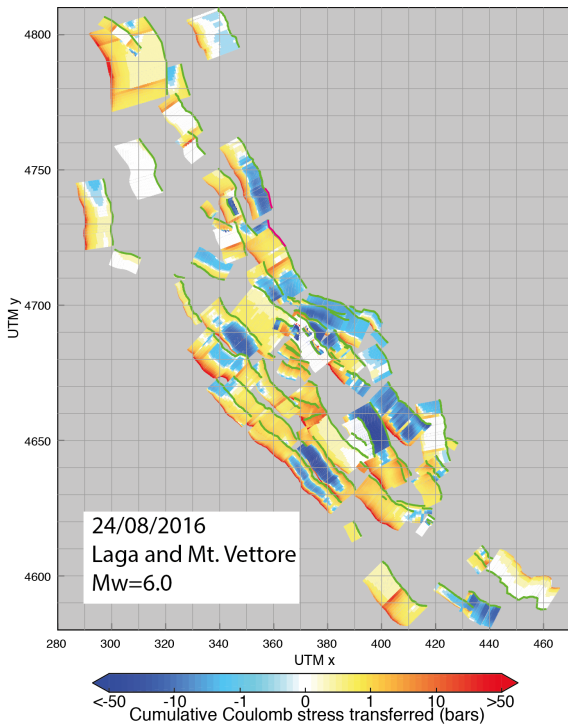

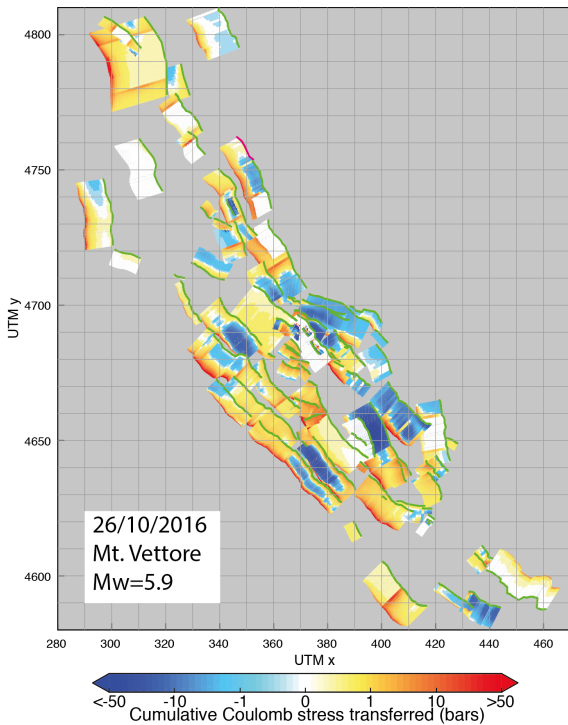

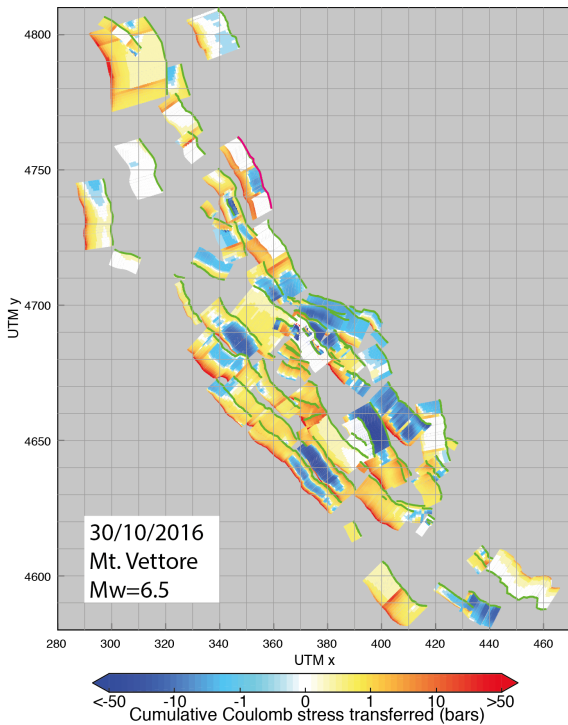

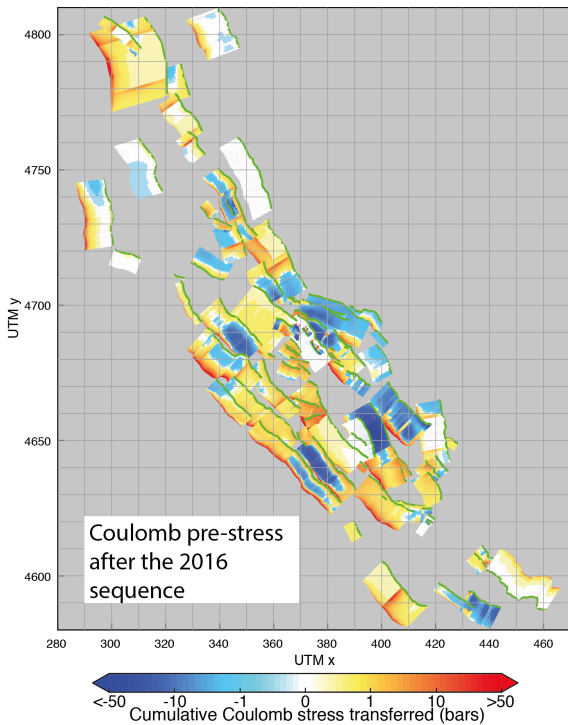

Supplement: Supplementary file 4 — Supplementary Data 2 [file 41467_2019_10520_MOESM4_ESM.pdf]

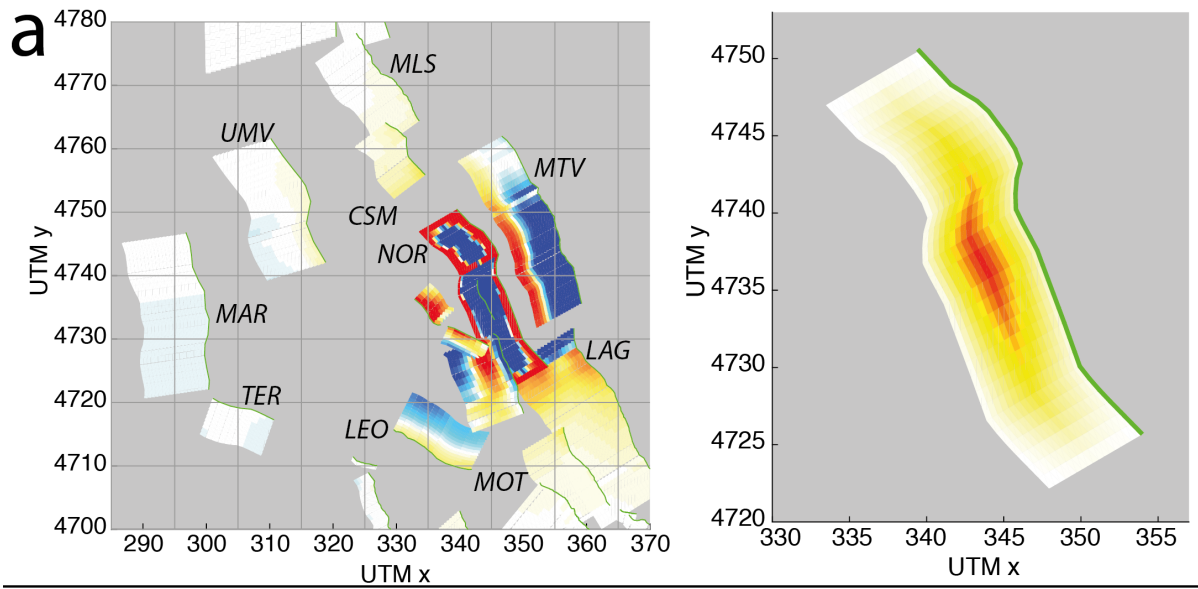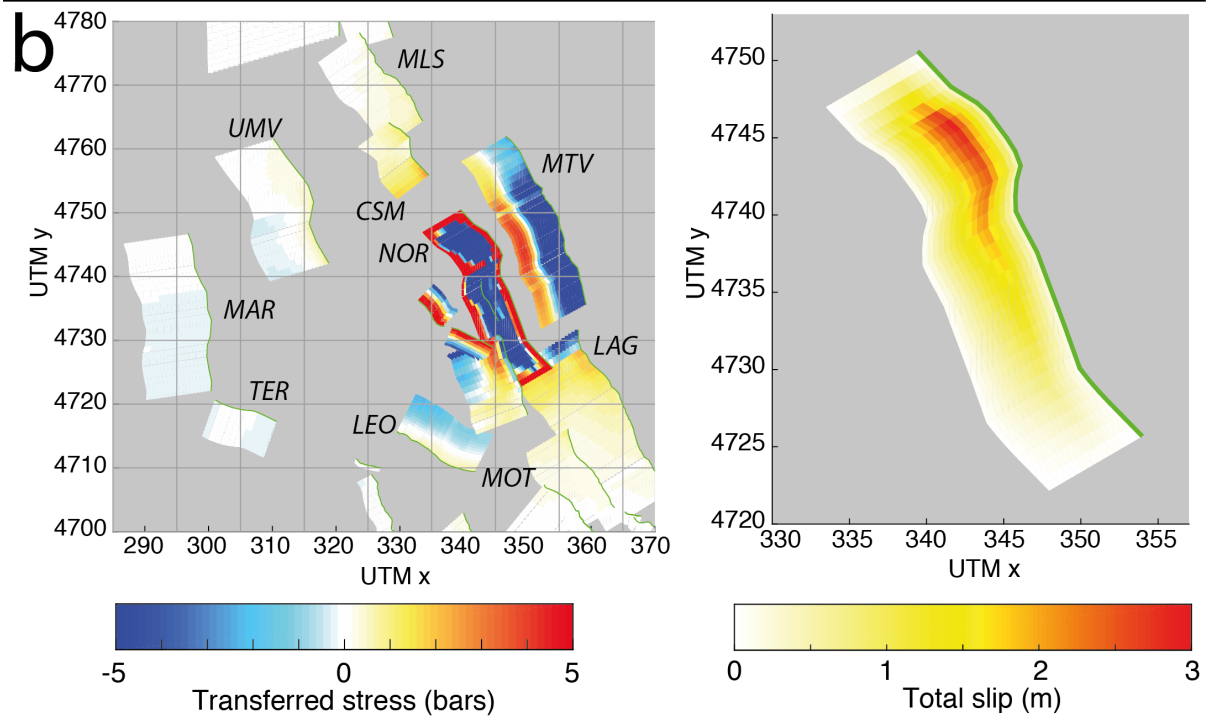

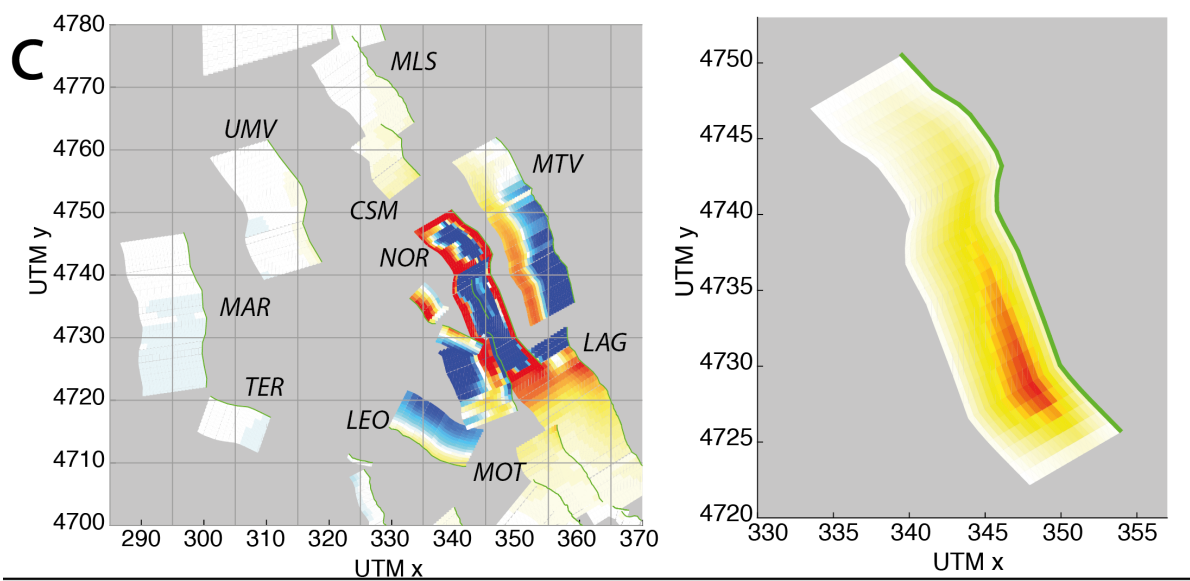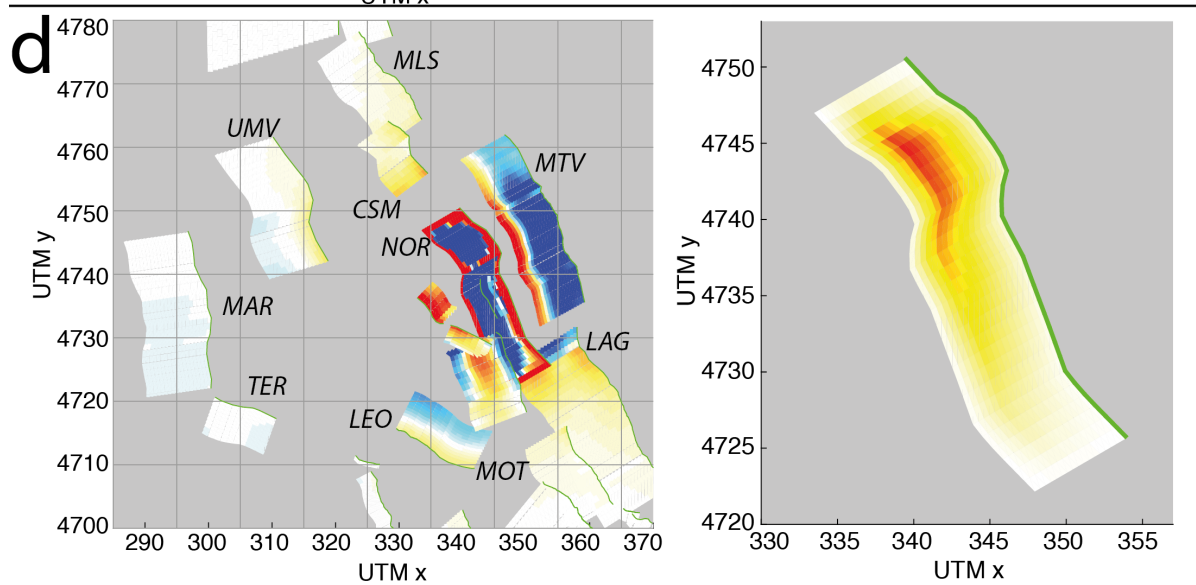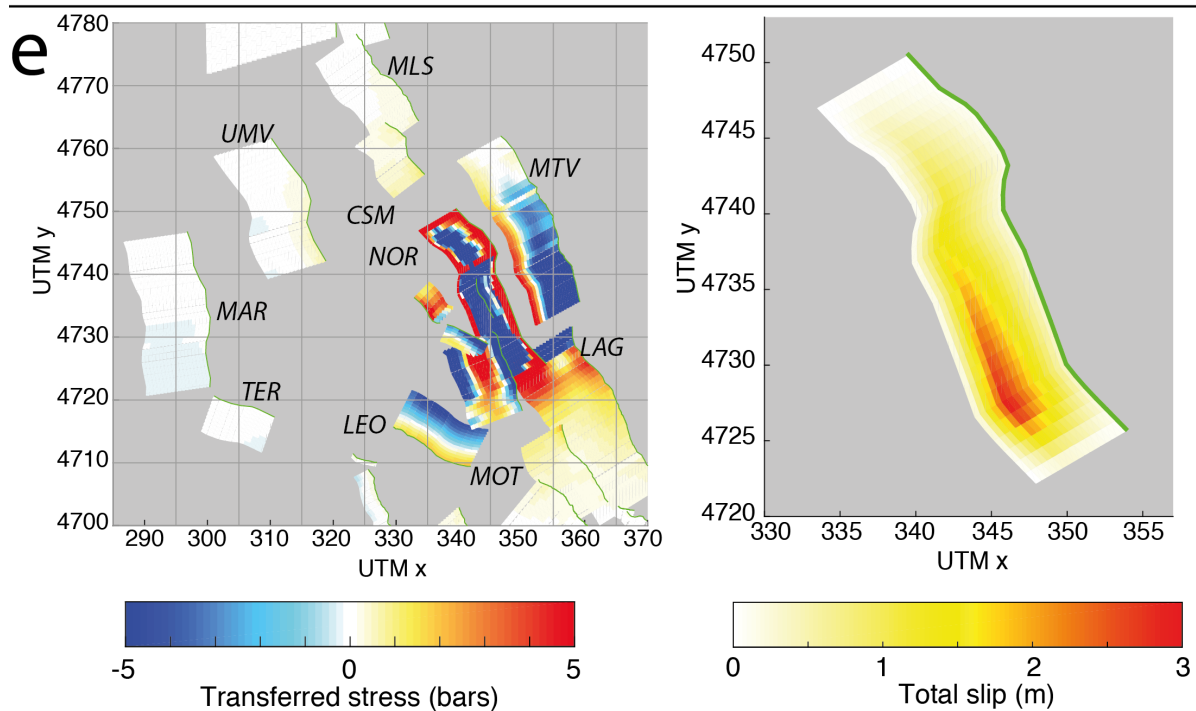

Supplement: Supplementary file 5 — Supplementary Data 3 [file 41467_2019_10520_MOESM5_ESM.pdf]
