# Supplementary material for: Coulomb pre-stress and fault bends are ignored yet vital factors for earthquake triggering and hazard
Source: Nat Commun. 2019 Jun 21;10:2744. doi: 10.1038/s41467-019-10520-6 (PMC6588554; doi:10.1038/s41467-019-10520-6)
Supplement: Supplementary file 7 — Supplementary Data 5 [file 41467_2019_10520_MOESM7_ESM.pdf]

Fault name	UTM x	UTM y	Slip vector trend (°)	Slip vector plunge (°)	15ka throw (m)	Strike (°)	Dip (°)
Assergi	386260	4696767				94	52
	369294	4701335				113	63
Barete	357115	4704065				150	40
	357129	4704092				145	41
	357132	4704123				157	45
	357164	4704063				145	40
	357170	4704064	251	39		148	40
	357221	4704038				149	42
	357448	4703575				161	39
	357450	4703589				160	36
	357453	4703572				150	33
	357458	4703560				152	36
	357463	4703538				161	40
	357466	4703521				156	39
	357468	4703513				148	38
	357469	4703558				153	37
	357515	4703485				145	37
	357520	4703468				149	42
	357530	4703414	234	41		148	42
	357538	4703448				148	42
	357544	4703439				147	42
	357608	4703356				128	38
	357610	4703346				132	39
	357624	4703330				128	39
	357631	4703322				120	38
	357643	4703317				120	37
	357647	4703316				122	37
	357656	4703308				127	42
	357662	4703301				127	37
	357675	4703294	208	37		125	38
	357687	4703291				122	38
	357692	4703285	212	39		120	38
	357702	4703288				120	38
	357709	4703286				124	39
	357717	4703280				123	37
	357726	4703268	206	39	11.12	119	39
	357733	4703274				123	38
	357740	4703271				123	38
	357750	4703266				118	38
	357755	4703254				121	36
	357764	4703252				120	36
	357775	4703249				123	37
	357794	4703253				102	34
	357811	4703254				118	34
	357818	4703252				118	35
	357823	4703251				110	36

	357827	4703255				104	31
	357843	4703264				118	38
	357851	4703262				128	36
	357862	4703260				115	35
	357918	4703277				104	54
	357946	4703269				131	35
	357955	4703266				127	34
	357969	4703272				86	40
	358076	4703105				137	59
	358082	4703084				173	66
	358095	4702902				159	49
	358096	4702879				160	52
	358096	4702939				183	64
	358113	4702779				156	51
	358129	4703028				190	55
	358144	4702711				140	48
	358172	4702645				152	54
	358180	4702622				150	52
	358186	4702601				151	49
	358212	4702422				135	44
Collebrincioni	368949	4698136				122	51
	368966	4698118				104	52
	368990	4698106				100	46
	369026	4698114				82	55
	369061	4698110				112	48
	369072	4698105				102	53
	369081	4698102				117	57
	369108	4698093	204	55		109	55
	369153	4698055				103	58
Costa San Martino	330915	4762283				117	54
	330933	4762234				137	62
	331045	4762143	215	57		118	57
	331105	4762079	213	56		131	60
	331109	4762072				127	57
	331117	4762067				119	58
	331130	4762055				124	57
	331137	4762050				109	58
	331145	4762043				117	56
	331152	4762037				117	54
	331160	4762026				119	55
	331167	4762020				111	57
Fontecchio	384531	4677933				116	76
	384552	4677923	184	77		123	78
	384595	4677890	202	60	3.6	114	61
	384607	4677886				114	58
	384628	4677878	201	65		117	60
	385590	4677445			3.9	115	50
Laga	365676	4717996	217	42		167	49

	365682	4717989				150	50
	365713	4717961				153	66
	365858	4717734				175	50
	365904	4717688				170	54
	365996	4717651	219	55		141	54
	366028	4717564				156	52
	366092	4717502			9.82	152	55
	366579	4716383	216	61	7.6	135	62
	366591	4716327	236	43		157	45
	366596	4716321				154	44
Martana	297370	4745014	193	46		147	49
	297388	4744988				148	62
Mt Le Scalette	330575	4769662				132	57
	330581	4769650				130	60
	330590	4769640				126	59
	330635	4769603				101	55
	330645	4769589				111	65
	332325	4766920				171	67
	332351	4766854				164	52
	332355	4766838	247	59		161	62
	332361	4766824				153	61
	332381	4766661				185	81
Ocre	367652	4682645				111	66
	367835	4682517				126	52
	367928	4682458				115	57
	368108	4682339				134	55
	368354	4682127				131	58
	368375	4682031				156	62
	368380	4682017				161	67
	368448	4681922				138	58
	368474	4681895				139	62
	368492	4681870				125	62
	368576	4681838				106	57
	368593	4681833				112	53
	368620	4681826				107	60
	368647	4681804				126	59
	368654	4681795				119	60
	368669	4681785				121	59
	368677	4681779				121	56
	368713	4681747			3.29	117	53
	368718	4681744				125	52
	368737	4681734				119	55
	368775	4681715				119	47
	368838	4681676				126	56
	368939	4681589				132	56
	368962	4681570				123	65
	368985	4681556				138	56
	369008	4681532				132	62

	369031	4681504				125	69
	369048	4681494				123	65
	369068	4681487				115	62
	369166	4681414				126	58
	369185	4681397				131	65
	369251	4681334				121	55
Paganica	371915	4694556			4.5	125	61
	373408	4691984	221				
	373844	4691449		60			
	373895	4691427	222				
	374000	4691332	216				
	374121	4691252	217	23			
	374157	4691194	222	54			
	375200	4690252					
	375574	4690196	226				
	376778	4690250	214				
Poggio Di Roio	368717	4685152				150	57
	368725	4685149				150	57
	368742	4685133				129	53
	368763	4685110				129	56
	368784	4685093				137	57
	368805	4685074				132	53
	368835	4685047			3.09	129	50
	368852	4685038				126	50
	368856	4685029				138	55
	368883	4684994				141	57
	368892	4684979			± 2.5 (eye estimat	137	57
	368911	4684956				141	54
	368933	4684929				139	56
	368939	4684923				133	58
	368948	4684911				131	61
	368973	4684896				129	58
	368986	4684884			± 2 (eye estimate	130	63
Roccapreturo	391205	4673905	200	52		108	53
	391217	4673901				151	60
	391236	4673884				106	57
	391258	4673866	225	63		151	64
	391280	4673837				125	64
	391292	4673824	195	56		114	58
	391314	4673817				124	60
	391327	4673775			4.3	140	61
	391367	4673730				152	70
	391377	4673704				139	69
	391398	4673676	231	66		148	66
	391428	4673634				142	70
	391453	4673608				117	71
	391483	4673546	217	63		152	67
	391501	4673529	208	73		118	73

391510	4673516			123	78
391531	4673497			137	63
391536	4673483			133	65
391559	4673463			127	67
391593	4673441			114	62
391614	4673434			131	67
391638	4673414			151	68
391649	4673386	212	61	139	64
391676	4673353			146	66
391682	4673347			128	72
391692	4673341			94	72
391707	4673338			119	70
391729	4673305			139	61
391734	4673258			162	65
391737	4673322			135	66
391743	4673305	195	71	129	73
391745	4673228			139	66
391760	4673204			135	60
391775	4673185			133	56
391809	4673121			154	66
391838	4673039			146	62
391852	4673019			114	59
391878	4673002			109	66
391909	4673001			108	53
391919	4672990			105	59
391933	4673000			110	60
391946	4672993			111	58
391967	4672991			109	56
391983	4672986			121	59
392058	4672970			102	47
392069	4672969			118	58
392088	4672951			146	60
392101	4672930			142	54
392123	4672914			114	63
392145	4672906			103	64
392152	4672903			105	57
392171	4672893			124	59
392217	4672850			120	64
392218	4672843			130	63
392229	4672823	186	62	107	63
392236	4672816			122	62
392254	4672807	180	60	90	61
392271	4672802	196	56	104	57
392285	4672788	297	56	102	57
392296	4672780			123	59
392359	4672737			115	64
392529	4672639	190	67	105	67
392549	4672629			109	68

392588	4672561			118	59
393532	4671947			134	53
393537	4671944			132	50
393541	4671946	230	48	133	49
393548	4671935			127	50
393554	4671926			128	55
393560	4671921			126	53
393569	4671913			125	54
393579	4671905			126	55
393592	4671893			127	60
393606	4671915			148	68
393665	4671836			134	57
393677	4671832	122	29	112	59
393695	4671811			135	58
393699	4671804			139	60
393706	4671794			138	62
393710	4671777	180	44	135	55
393711	4671781			147	60
393718	4671776	177	36	137	54
393729	4671762			139	55
393740	4671756	239	55	147	55
393753	4671735			145	55
393768	4671701			146	56
393773	4671576			160	56
393773	4671532	197	57	152	61
393781	4671613			151	54
393781	4671511			154	63
393781	4671481			148	60
393782	4671621			168	61
393784	4671665			184	63
393785	4671635			183	62
393788	4671465			153	62
393791	4671445			156	58
393799	4671404			156	59
393807	4671384			154	57
393811	4671363			155	56
393822	4671353	264	44	140	55
393835	4671340			140	57
393838	4671301			132	60
393839	4671318			143	57
393954	4671238			122	54
393971	4671229			121	57
393977	4671222			116	58
393995	4671211			142	46
394004	4671182			138	57
394035	4671143			135	54
394279	4671101			133	50
394291	4671091			142	43

	394311	4671082				130	47
	394317	4671055				125	46
	394318	4671073				144	50
	394322	4671035				131	51
	394376	4670955				120	50
	394420	4670926				112	57
	394429	4670922				122	53
	394467	4670916	224	48		113	53
	394472	4670917				124	54
	394484	4670904				132	49
	394486	4670888	242	50		125	53
	394500	4670871				113	57
	394531	4670857				122	48
	394545	4670849	195	52		106	54
	394638	4670848				94	55
	394670	4670859				91	55
	394783	4670854	230	44		97	54
	394793	4670854	202	52		96	53
	394810	4670857	210	51		87	55
	394828	4670855				105	62
	394845	4670849				102	56
	394858	4670844	210	56		94	58
	394882	4670839				100	60
	394955	4670814				120	49
	394971	4670805				131	42
	395007	4670708	247	39		117	47
	395029	4670692	261	43		110	61
	395036	4670693				115	58
	395061	4670681				111	61
	395075	4670677				123	60
	395084	4670668				114	57
	395097	4670666				107	52
	395122	4670670				101	57
	395156	4670669				98	50
	395169	4670666				90	50
	395189	4670661				95	52
	395215	4670648				94	50
	395229	4670622				106	42
	395230	4670650				103	49
Scanno	406796	4643001			4.5	136	55
	406678	4642989				128	42
	406929	4642938				124	47
	407316	4642684				138	56
	407332	4642639				143	62
	407413	4642561	224	58		121	60
	407429	4642555				129	50
	407451	4642537				137	53
	407455	4642534				136	53

	407462	4642521				135	57
	407467	4642516				135	54
	407503	4642484				142	51
	407511	4642459				134	51
	407569	4642427				134	46
	407571	4642414				120	53
	407580	4642390				131	56
	410662	4637993				138	43
Sulmona	408489	4663788				129	53
	408508	4663772				126	56
	408518	4663761				128	51
	408556	4663742				108	51
	408622	4663729				125	51
	408641	4663710				126	53
	408662	4663705				107	46
	408675	4663707				91	51
	408723	4663692				108	46
	411862	4660282				152	53
Terni	308308	4718570	251	35		126	41
	308406	4718515				99	47
	308610	4718418				106	64
	308626	4718418				87	42
Vettore	355145	4747661				199	65
	355146	4747705				164	59
	355146	4747643				184	57
	355149	4747681	235	61		162	61
	355152	4747619	214	72		154	71
	355159	4747608	233	59		142	61
	355166	4747574				144	65
	355178	4747564			5.2	151	56
	355184	4747556				141	59
	355190	4747542				172	67
	355198	4747521				138	64
	355215	4747482	234	68		157	68
	355220	4747468				141	63
